# Dissecting the causal polymorphism of the *Lr67res* multipathogen resistance gene

**DOI:** 10.1093/jxb/erae164

**Published:** 2024-05-15

**Authors:** Ricky J Milne, Katherine E Dibley, Jayakumar Bose, Adnan Riaz, Jianping Zhang, Wendelin Schnippenkoetter, Anthony R Ashton, Peter R Ryan, Stephen D Tyerman, Evans S Lagudah

**Affiliations:** CSIRO Agriculture and Food, Canberra, ACT 2601, Australia; CSIRO Agriculture and Food, Canberra, ACT 2601, Australia; Australian Research Council Centre of Excellence in Plant Energy Biology, School of Agriculture, Food and Wine, University of Adelaide, Urrbrae, SA 5064, Australia; School of Science and Hawkesbury Institute for the Environment, Western Sydney University, Richmond, NSW 2753, Australia; CSIRO Agriculture and Food, Canberra, ACT 2601, Australia; AgriBio, Centre for AgriBioscience, Bundoora, VIC 3083, Australia; CSIRO Agriculture and Food, Canberra, ACT 2601, Australia; Plant Breeding Institute, School of Life and Environmental Sciences, University of Sydney, Cobbitty, NSW 2570, Australia; CSIRO Agriculture and Food, Canberra, ACT 2601, Australia; CSIRO Agriculture and Food, Canberra, ACT 2601, Australia; CSIRO Agriculture and Food, Canberra, ACT 2601, Australia; Australian Research Council Centre of Excellence in Plant Energy Biology, School of Agriculture, Food and Wine, University of Adelaide, Urrbrae, SA 5064, Australia; CSIRO Agriculture and Food, Canberra, ACT 2601, Australia; Sichuan Agricultural University, China

**Keywords:** Adult plant resistance, biotrophic pathogens, multipathogen resistance, non-canonical resistance gene, STP13/Lr67, wheat rust

## Abstract

Partial resistance to multiple biotrophic fungal pathogens in wheat (*Triticum aestivum* L.) is conferred by a variant of the *Lr67* gene, which encodes a hexose–proton symporter. Two mutations (G144R and V387L) differentiate the resistant and susceptible protein variants (Lr67res and Lr67sus). Lr67res lacks sugar transport capability and was associated with anion transporter-like properties when expressed in *Xenopus laevis* oocytes. Here, we extended this functional characterization to include yeast and *in planta* studies. The *Lr67res* allele, but not *Lr67sus*, induced sensitivity to ions in yeast (including NaCl, LiCl, and KI), which is consistent with our previous observations that *Lr67res* expression in oocytes induces novel ion fluxes. We demonstrate that another naturally occurring single amino acid variant in wheat, containing only the Lr67^G144R^ mutation, confers rust resistance. Transgenic barley plants expressing the orthologous *HvSTP13* gene carrying the G144R and V387L mutations were also more resistant to *Puccinia hordei* infection. NaCl treatment of pot-grown adult wheat plants with the *Lr67res* allele induced leaf tip necrosis and partial leaf rust resistance. An Lr67res-like function can be introduced into orthologous plant hexose transporters via single amino acid mutation, highlighting the strong possibility of generating disease resistance in other crops, especially with gene editing.

## Teaser

A wheat multipathogen resistance gene that encodes a sugar transporter variant lacks a sugar transport function, but, in addition to disease resistance, confers ion-related phenotypes in yeast and plants.

## Introduction

Wheat provides an important source of calories and protein for human consumption, and global wheat production reached 771 Mt in 2021 (FAO, 2023). Fungal diseases pose a constant threat to wheat production and represent a limiting factor to yield improvement. In addition to affecting wheat, fungal diseases decrease yields of many other crops grown worldwide. Disease resistance conferred by plant-encoded genes is an environmentally safe strategy for disease control. Immune receptor resistance genes (*R* genes) make up the majority of wheat-encoded rust resistance and often confer strong resistance triggered by a hypersensitive response to single pathogen races. Distinct from these *R* genes, a number of unique non-canonical resistance genes in wheat (*Lr34*, *Lr46*, and *Lr67*) are able to confer partial resistance to multiple pathogens in a non-race-specific manner (Singh *et al.,* 1998; Krattinger *et al.,* 2009; Kolmer *et al.*, 2015; Moore *et al.*, 2015). Of these three genes, *Lr34* has been widely deployed in commercial wheats for decades and is yet to be overcome by pathogens, signifying the durability of this resistance (Ellis *et al.*, 2014). Although less widely deployed than *Lr34*, resistance by *Lr67* has also not been overcome by pathogens. Importantly, in the context of this study, some wheat disease resistance genes including *Lr34* and *Lr67* have cross-species functionality and, when expressed as a transgene in other species, they confer resistance to pathogens that do not colonize wheat (Sucher *et al.*, 2017; Milne *et al.*, 2019). This can be incredibly useful for generating novel resistance in crops where germplasm and/or genetic resources are limited or not well developed, or additionally where rapid pathogen evolution occurs to overcome prevailing major resistance genes in commercial cultivars. Another scenario where genes such as *Lr34*/*Lr67* may be useful to improve pathogen resistance is in smallholder crops where fungicides are too expensive for growers. In the case of *Lr67*, this gene encodes a Sugar Transport Protein 13 (STP13) variant possessing two amino acid changes from the wild-type *Lr67sus* allele (G144R and V387L), and direct orthologues of this gene are conserved in all plants (Moore *et al.*, 2015). Further, the single nucleotide change responsible for mutation of a highly conserved glycine to an arginine (G144R) appears to be sufficient to confer resistance when introduced into the orthologous *STP13* of other species (Gupta *et al.*, 2021; Skoppek *et al.*, 2022), representing a promising target for mutagenesis and/or gene editing.

Systems to characterize immune receptor-type *R* genes are numerous, well established, and useful for demonstrating *R* gene function via a detectable phenotype when plant-encoded R proteins and pathogen-encoded avirulence (Avr) proteins interact. A cell death phenotype or hypersensitive response is usually triggered by such interactions and is routinely detected in various expression systems such as leaf protoplasts and *Nicotiana benthamiana* leaves (Saur *et al.*, 2019; Xi *et al.*, 2021). However non-canonical resistance proteins such as Lr67 and others that reduce fungal development in the absence of a hypersensitive response, given their distinct molecular mechanisms from immune receptor R proteins, prove to be less straightforward to develop simple assays demonstrating function. Further, such functional assays would prove to be useful in the context of transferring *Lr67res*-like resistance into other crops via STP13 orthologues prior to undertaking lengthy stable plant transformation. Whilst some systems do exist, they are often specialized or specific to certain crop pathosystems and may not be applicable across plant species. A function associated with the wheat Lr67res protein, characterized by enhanced anion fluxes, was recently demonstrated using the specialized heterologous expression system, *Xenopus laevis* oocytes (Milne *et al.,* 2023). Interestingly, race-specific *R* genes have also been associated with altered ion fluxes in plant cells, albeit via a mechanism distinct from that of Lr67res-associated ion transport. In this case, calcium fluxes lead to a hypersensitive response (Bi *et al.*, 2021; Jacob *et al.*, 2021).

In this study, we aimed to further our understanding of the *Lr67res* multipathogen resistance gene. Previous demonstration of function made use of the *Xenopus* oocyte system which is highly specialized; therefore, we aimed to develop a functional assay not requiring specialized equipment or techniques as part of this study. Here, we demonstrate that an Lr67res ion-associated phenotype observed in *Xenopus* oocytes can be extrapolated to other biological systems—yeast and wheat. Curiously, elevated Na^+^ content was detected in Lr67res oocytes despite anion currents being detected along with increased ^36^Cl^−^ uptake, which led us to develop a yeast screening assay based on growth on media supplemented with NaCl, producing a phenotype that differentiated between *Lr67sus* and *Lr67res* alleles. Further, similar observations were able to be made using multiple wheat genotypes where NaCl-induced leaf tip necrosis was observed in those carrying *Lr67res*, and partial resistance to *Puccina triticina* occurred in the presence of this leaf tip necrosis. The findings of this study indicate that Lr67res ion-associated phenotypes are not limited to *Xenopus* oocytes and also appear to be relevant in the context of disease resistance. Moving forward, these could prove valuable tools to assess for resistance-like phenotypes of synthetic candidate resistance genes prior to lengthy plant transformation experiments.

## Materials and methods

### Constructs for yeast expression

The coding sequence of *Lr67sus* in the pENTR1A entry vector (Life Technologies, Mulgrave, VIC, Australia) as previously described (Milne *et al.*, 2023) was used as a template to introduce site-directed mutations. Lr67sus G144A, G144C, G144D, G144K, G144S, and G144W were produced using the primers listed in Supplementary Table S1, designed by QuikChange (https://www.genomics.agilent.com/primerDesignProgram.jsp) using the protocol as described for *HvSTP13* (Milne *et al.*, 2019). Each gene variant was then subcloned into pDR196 using the *Eco*RI and *Xho*I restriction sites. Gene sequences were confirmed by Sanger sequencing (AGRF, Westmead, NSW, Australia).

For heterologous expression in yeast, *Lr67* alleles, *Lr34* alleles, and *Lr67res* loss-of-resistance (LOR) mutants, as described previously (Milne *et al.*, 2023), in either the pDR195, pDR196 (Rentsch *et al.*, 1995), or pDR196T (Milne *et al.*, 2019) vectors, were used (Supplementary Table S2). *AtSTP13* (*Xho*I and *Not*I), *SbSTP13* (*Eco*RI and *Xho*I), *XylE* (*Eco*RI and *Xho*I), *AtHKT1* (*Not*I and *Bam*HI), and *TmClC-0* (*Spe*I and *Xho*I) cDNAs synthesized by Integrated DNA Technologies (Singapore Science Park II, Singapore) with flanking restriction sites as indicated were cloned into pDR195 or pDR196. *GLUT1* (*Not*I and *Bam*HI) synthesized by GeneArt (ThermoFisher, Scoresby, VIC, Australia) was cloned into pDR195. Constructs are detailed in Supplementary Table S2. Site-directed mutants for G144R (or equivalent) were produced after cloning into pENTR1A using the primers in Supplementary Table S1 and the protocol above, and then subcloned using flanking restriction sites into pDR195 or pDR196. The G144E variant was synthesized and sub-cloned into pDR196 by Twist Bioscience (San Francisco, CA, USA). FLAG-tagged *Lr67* alleles in p426ADH1 as described by Moore *et al*. (2015) were subcloned into pDR196 using *Eco*RI and *Sal*I restriction sites. Yeast expression constructs were transformed into yeast as described (Dohmen *et al.*, 1991), and at least three colonies were selected as independent transformation events (biological replicates). Colonies were cultured in synthetic dropout medium lacking uracil (SDura^−^; 6.72 g l^–1^ yeast nitrogen base with ammonium sulfate, 0.96 g l^–1^ yeast synthetic dropout medium without uracil, 2% w/v maltose), plasmids were rescued from yeast as described using the QIAprep Miniprep Kit (QIAGEN, Chadstone Centre, VIC, Australia; User developed protocol: isolation of plasmid DNA from yeast), transformed to *Escherichia coli*, and sequences were confirmed by Sanger sequencing (AGRF). Yeast strains harbouring constructs of *Lr67* alleles driven by the yeast *ADH1* promoter in p426ADH1 were used as described (Moore *et al*., 2015).

### Yeast plate assays

EBY.VW4000 yeast (Wieczorke *et al.*, 1999) (incapable of hexose uptake) and B31 yeast (Bañuelos *et al.*, 1998) (incapable of Na^+^ efflux) were transformed using the polyethylene glycol (PEG) 1000 protocol (Dohmen *et al.*, 1991). Transformed yeasts were cultured in liquid SDura^−^ medium to early logarithmic phase (OD_600_ of 0.8–1.0) and standardized to an OD_600_ of 0.8. A decimal dilution series of yeast suspension (3 µl) was spotted onto solid (15 g l^–1^ agar) SDura^−^ medium ±salts as indicated in the figure legends. Glucose was used in place of maltose in complementation assays and in B31 growth assays. Spot plates were incubated at 30 °C for 2 d (~48 h) before photographing. Three biological replicates (three independent colonies from a transformation plate) of each construct were tested, and spot plate experiments were replicated at least twice. CY162 yeast (Ko and Gaber, 1991) (incapable of K^+^ uptake) growth assays were performed as above using AP medium (Rodríguez-Navarro and Ramos, 1984) with 2% glucose and the indicated concentrations of KCl, and grown at 30 °C for 6 d. Medium lacking uracil and tryptophan was used in yeast co-expression experiments, and the pDR196T vector was used for tryptophan selection (Milne *et al.*, 2019).

### Field rust resistance screening of the *Lr67* transmembrane region 4 variant

Naturally occurring *Lr67* variants containing only the transmembrane region 4 single nucleotide polymorphism (SNP), encoding the G144R mutation, were crossed from the progenitor donor wheat landrace, AUS4793, to the susceptible cultivar, Avocet S. Homozygous F_3_ sib selections with G144R were grown in the field alongside Avocet S (*Lr67sus*) and Avocet*+Lr67res* (G144R, V387L), inoculated with stripe rust, and disease severity was assessed on flag leaves of adult plants in accordance with previous phenotyping protocols (Moore *et al*., 2015).

### Production of transgenic wheat

Wheat cv. Fielder transgenics were used as described previously (Moore *et al*., 2015). The construct described previously—a 7133 bp genomic fragment from the Thatcher*+Lr67res* genotype containing the 1318 bp native promoter, genomic *Lr67res* sequence, and 1512 bp of the 3'-untranslated region (UTR) cloned into pVecNeo—was used to transform cv. Stewart durum wheat. An *Agrobacterium tumefaciens*-mediated transformation protocol licensed by Japan Tobacco was utilized (Richardson *et al.*, 2014; Ishida *et al.*, 2015). Transformants were genotyped with a KASPar marker (Supplementary Table S1) and the KASP Master mix (KBiosciences/LGC Genomics, Teddington, Middlesex, UK).

### Cabinet-grown wheat NaCl treatment and rust inoculation

Wheat near-isogenic lines of cv. Thatcher (which carries the wild-type *Lr67sus* allele) and Thatcher*+Lr67res* (Dyck and Samborski, 1979; Dyck *et al.*, 1994) were subjected to salt treatment at the adult stage of development. Two plants were grown per 15 cm diameter pot in a soil mixture of 50% compost, 25% sand, and 25% perlite under an 18 h day/6 h night light cycle with a light intensity of 300–350 µmol m^–2^ s^–1^ at temperatures of 22 °C/18 °C, respectively. Pots were freely draining for the duration of the experiment. Six pots of each genotype were randomly assigned for control or salt treatment (12 plants per treatment per genotype). All pots were watered with a quarter strength Hoagland’s solution (Hoagland Modified Basal Salt Mixture, Phytotech Labs, Lenexa, KS, USA) 1 week prior to salt treatment. Half the pots were then watered with 25 mM NaCl+1.67 mM CaCl_2_; a 15:1 ratio of Na^+^:Ca^2+^ was used as described previously (Cramer, 2002; Hussain *et al.*, 2004) in half-strength Hoagland’s solution at anthesis, and then with 50 mM NaCl+2.5 mM CaCl_2_ half-strength Hoagland’s solution 6 d later, whilst control pots were watered with half-strength Hoagland’s solution only. For each treatment, 350 ml of nutrient solution was applied per pot, and pots were flushed with 700 ml of water 24 h after treatment. Plants were watered to field capacity 24 h prior to treatment. Flag leaves and penultimate leaves were photographed 11 d after initial NaCl treatment. The 25 mM NaCl followed by 50 mM NaCl treatment regime was selected as a mild treatment level as previously determined in wheat (Husain *et al.*, 2004).

A separate experiment was performed as above with cv. Thatcher and Thatcher*+Lr67res* wheat where eight pots of three plants per pot were grown and half underwent the same control or salt treatment. Thereafter, 4 d post-second salt treatment, all plants were inoculated with *P. triticina* 19ACT07_01 (Pathotype 104-1,3,4,5,6,7,9,10,12+Lr37). After a 2 min heat shock treatment at 42 °C, spores of *P. triticina* were mixed with talc powder and spread evenly over flag leaves by hand. Humidity (∼100%) was maintained by incubating plants in a sealed container under the same growth conditions as above for 48 h post-inoculation. The midpoint of inoculated flag leaves was harvested at 5 days post-inoculation (dpi) for microscopic histological assessments to determine representative infection site sizes at described (Ayliffe *et al.*, 2013) by staining chitin present in fungal structures with wheat germ agglutinin conjugated to fluorescein isothiocyanate (WGA–FITC). Microscopic images were photographed using an Axio Imager Z2 microscope and ZEISS ZEN software (Zeiss, North Ryde, NSW, Australia). Inoculated flag leaves were harvested 9 dpi, photographed, and ImageJ was used to quantify the infected leaf area.

NaCl induction of leaf tip necrosis was also examined on two cv. Fielder+*Lr67res*-independent transgenic events of the T_6_ generation (26a-14 and 2b-5) and Fielder-negative segregants lacking the transgene (Moore *et al*., 2015), and two cv. Stewart+*Lr67res*-independent transgenic events of the T_3_ generation (P205-10, P205-17) and Stewart-negative segregants lacking the transgene. Treatment with NaCl was performed as described above, with three plants grown per pot, and leaves were imaged 7 d post-second treatment.

### Production and rust inoculation of transgenic barley

A modified version of the *HvSTP13* genomic sequence (HORVU4Hr1G067450.1) incorporating G144R and V387L mutations was synthesized by Epoch Biolabs (Sugar Land, TX, USA), including the 1503 bp promoter sequence upstream of the start codon, the 1660 bp 3'-UTR sequence after the stop codon, and flanked by *Not*I restriction sites. This genomic fragment was inserted into the pVec8 vector and transformed to *A. tumefaciens* strain AGL0, which was used to transform barley cv. Golden Promise embryos as described (Harwood, 2014). Transformants were genotyped with a KASPar marker (Supplementary Table S1) and the KASP Master mix (KBiosciences/LGC Genomics, Teddington, Middlesex, UK).

Plant growth and inoculation were performed as described (Milne *et al.*, 2019). Transgenic barley plants of the T_2_ generation and negative segregants lacking the transgene (−sibs) were housed in a growth cabinet under a 16 h day/8 h night light cycle with a light intensity of 300–350 µmol m^–2^ s^–1^ at a constant temperature of 13 °C. After a 2 min heat shock treatment at 42 °C, spores of the *Pucchinia hordei* pathotype 5457P+ (Singh *et al.*, 2018) (kindly provided by the Plant Breeding Institute, Cobbitty, NSW, Australia) were mixed with talc powder and sprayed over barley plants at the five-leaf stage. Humidity (∼100%) was maintained by incubating plants in a sealed container at 20 °C for 72 h post-inoculation. The youngest fully expanded leaves were harvested 8 dpi, photographed, and prepared for microscopic analysis. Microscopic histological assessments were used to determine representative infection site sizes at 8 dpi as described (Ayliffe *et al.*, 2013) by staining chitin present in fungal structures with WGA–FITC. Microscopic images were photographed using an Axio Imager Z2 microscope and ZEISS ZEN software (Zeiss, North Ryde, NSW, Australia).

### Accession numbers

NCBI accession numbers of sequences used in this study are as follows unless specified. *Lr67sus*, MV144992.1; *Lr67res*, KR604817.2; *AtHKT1*, NM_117099.6; *AtSTP13*, AJ344338.1; *GLUT1*, NM_006516.4; *HvSTP13*, MK409638.1; *Lr67res* genomic clone (MK425206); *HvSTP13* genomic clone HORVU4Hr1G067450.1/ HORVU.MOREX.r3.4HG0396130 (Ensembl Plants); *Lr34sus*, HL100988.1; *Lr34res*, XM_044586927.1; *SbSTP13*, XM_002465591.2; *TmCLC-0*, X56758.1; and *XylE*, AAA79016.1 (European Nucleotide Archive).

## Results

### Development of a yeast phenotypic screen based on the ion-associated function of Lr67res

**Fig. 1.**
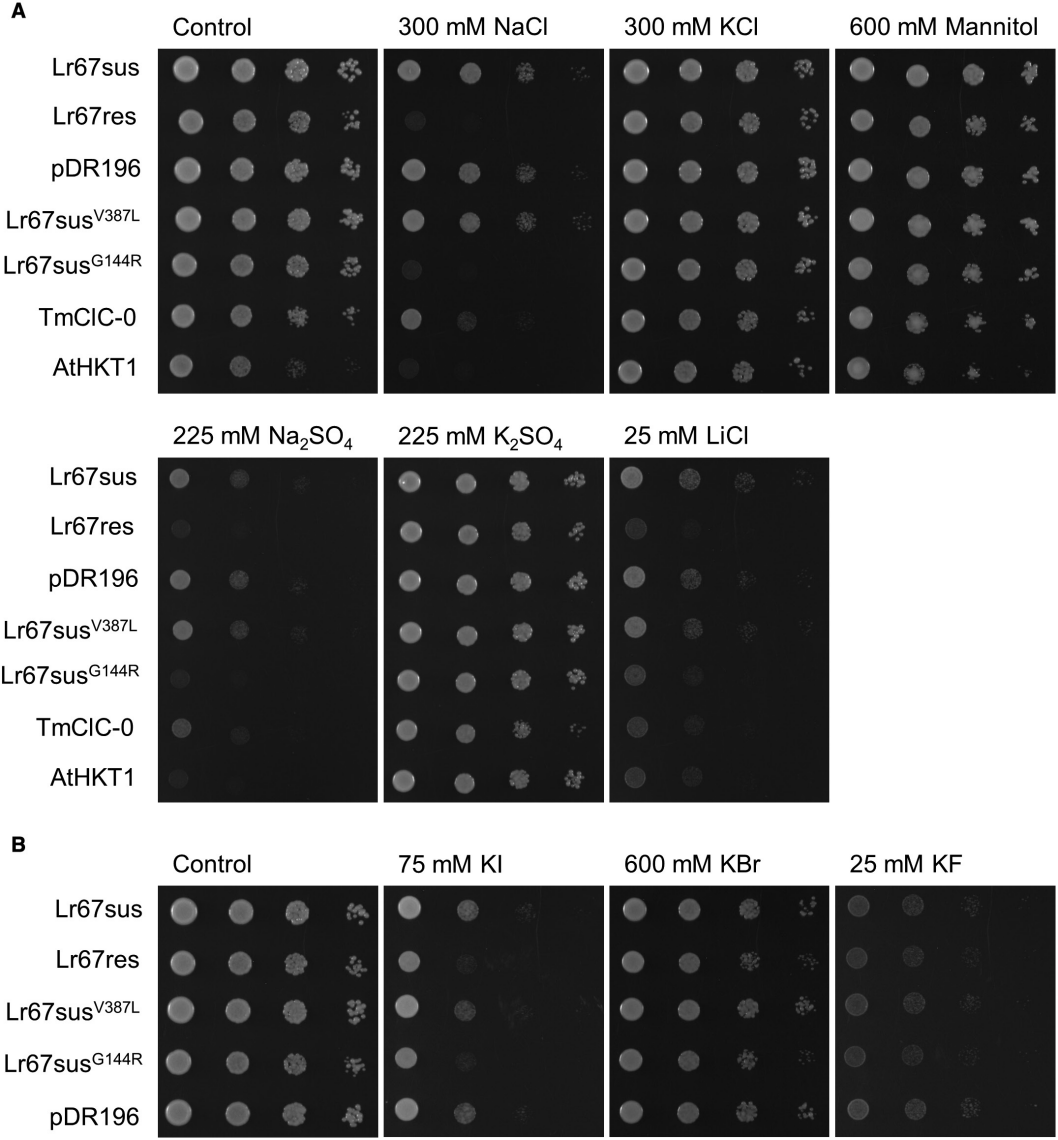
Assessment of Lr67res-induced ion sensitivity in yeast. Decimal dilution series of EBY.VW4000 yeast, from an OD_600_ of 0.8, transformed with *Lr67* alleles, *Lr67* site-directed single mutants, pDR196 empty vector, the *Torpedo marmorata* chloride channel, *TmClC-0*, and the Arabidopsis high affinity K^+^ transporter, *AtHKT1* grown on medium containing different salts to screen for (A) cation sensitivity or (B) anion sensitivity. Mannitol was included as an osmotic control. Images are representative of three biological replicates (independently transformed colonies).

Since previous studies showed that *Lr67res* expression in *X. laevis* oocytes was associated with enhanced ion fluxes, accumulation of Na^+^ and elevated Cl^−^ uptake (Milne *et al.*, 2023), we investigated phenotypic screens in yeast based on sensitivity to NaCl. Yeast harbouring various *Lr67* alleles with and without additional site-directed mutations were grown on solid medium containing a range of salts. On medium lacking Na^+^ and containing 2 mM Cl^−^, the growth of EBY.VW4000 yeast cells transformed with *Lr67res*, *Lr67sus*, and the empty vector control (pDR196) was similar. In the presence of an additional 300 mM NaCl, growth of Lr67res yeast was considerably slower than that of yeast transformed with *Lr67sus* and the empty vector (Fig. 1A). Yeast expressing the *Arabidopsis thaliana High-affinity K+ transporter 1* (*AtHKT1*), an Na^+^ selective transporter gene (Uozumi *et al.*, 2000), was also sensitive to 300 mM NaCl (Fig. 1A), whereas expression of *Lr67res* in B31 yeast cells (unable to efflux Na^+^) (Bañuelos *et al.*, 1998) increased its sensitivity to only 50 mM NaCl (Supplementary Fig. S1A). These results are consistent with *Lr67res* expression in yeast being associated with increased Na^+^ or Cl^−^ uptake. Compared with Lr67sus, Lr67res also retarded yeast growth on medium supplemented with Na_2_SO_4_ and LiCl, but not on medium supplemented with KCl, K_2_SO_4_, or mannitol (Fig. 1A). Interestingly, EBY.VW4000 yeast harbouring the chloride channel TmClC-0 (Jentsch *et al.*, 1990) were sensitive to NaCl and Na_2_SO_4_ (both to a lesser extent than Lr67res), and to LiCl (Fig. 1A).

Given the lack of toxicity associated with KCl, a range of K^+^ salts were used to screen for anion-induced toxicity. Growth of Lr67res yeast was similar to that of controls on medium containing KBr or KF but it was considerably slower on medium supplemented with KI, presumably due to I^–^ anion uptake which is known to be toxic to yeast (Greaves *et al.*, 1928) (Fig. 1B). Sensitivity of yeast to NaCl was correlated with the level of *Lr67res* expression since growth was inhibited more when the strong plasma membrane H^+^-ATPase 1 (*PMA1*) promoter was used to drive *Lr67* expression, and less when the weaker alcohol dehydrogenase 1 (*ADH1*) promoter was used to drive expression (Haresh Liya *et al.*, 2023) (Supplementary Fig. S1B). Additionally, N- and C-terminal tags on Lr67res reduced yeast sensitivity to NaCl, which demonstrates that the sensitivity is caused by the Lr67res protein (Supplementary Fig. S1C), assuming that correct plasma membrane targeting still occurs. Lr67res was unable to rescue the K^+^ uptake-deficient yeast strain CY162 (Ko and Gaber, 1991) (Supplementary Fig. S1D) which is consistent with previous results from oocyte studies showing that Lr67res is unlikely to transport K^+^ (Milne *et al.*, 2023).

### Lr67res loss-of-resistance mutants reduce yeast NaCl sensitivity

**Fig. 2.**
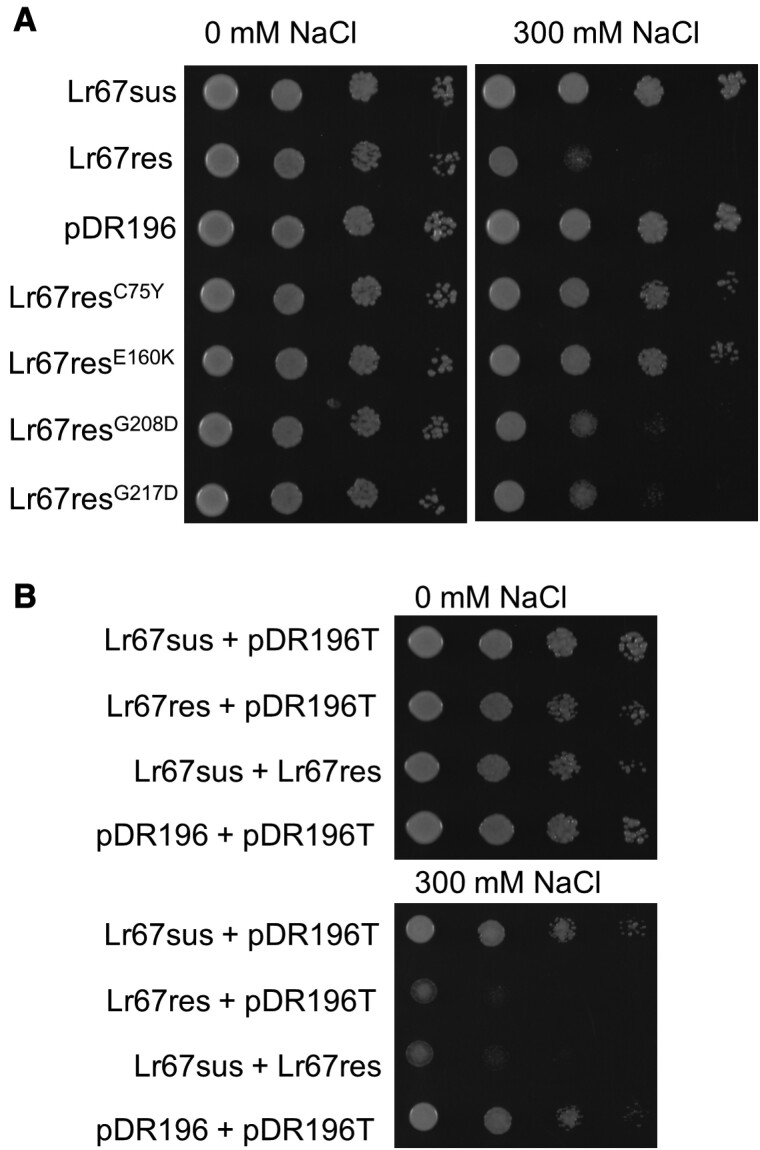
Lr67res loss-of-resistance (LOR) mutants exhibit reduced NaCl sensitivity, and co-expression of Lr67 alleles does not reduce NaCl sensivity. (A) Decimal dilution series of EBY.VW4000 yeast, from an OD_600_ of 0.8, transformed with *Lr67sus*, *Lr67res*, pDR196 empty vector, or *Lr67* LOR mutants grown on medium supplemented with NaCl as indicated. (B) Decimal dilution series of EBY.VW4000 yeast co-transformed with *Lr67* alleles and pDR196/pDR196T empty vectors. Images are representative of three biological replicates (independently transformed yeast colonies).

Previous studies have revealed that wheat lines carrying *Lr67res* exhibit partial resistance to rusts and other pathogens, accompanied by leaf tip necrosis in field conditions. Several *Lr67res* mutations induced in these lines resulted in a loss of disease resistance and absence of leaf tip necrosis. Among these LOR mutants, several had single amino acid changes in Lr67res while preserving the G144R and V387L substitutions, and these mutants also lacked sugar transport capacity (Spielmeyer *et al.*, 2013; Moore *et al.*, 2015; Milne *et al.*, 2023). The Lr67res C75Y, E160K, G208D, and G217D mutants were analysed in this study. The cDNAs of these LOR mutants were expressed in yeast to determine whether the additional amino acid changes affected the sensitivity of yeast to NaCl. Of the four mutants tested, growth of Lr67res^C75Y^and Lr67res^E160K^ yeast on 300 mM NaCl was similar to that of Lr67sus yeast, whereas Lr67res^G208D^ and Lr67res^G217D^ yeast gave an intermediate sensitivity to NaCl between that of Lr67res and Lr67sus (Fig. 2A). The C75Y and G208D mutants had previously been characterized in *X. laevis* oocytes, and the relative magnitudes of currents observed in those experiments are consistent with this observed relative sensitivity to NaCl for these mutations in yeast. In oocytes, reduction in current magnitudes for the C75Y mutation was also similar to that for Lr67sus (Milne *et al.*, 2023). Given the previously proposed dominant negative interference hypothesis as a mode of action for Lr67res disease resistance (Moore *et al*., 2015), *Lr67res* co-expression with *Lr67sus* was also tested in yeast for any impact on NaCl sensitivity. *Lr67res* NaCl sensitivity was maintained, regardless of co-expression with *Lr67sus* or an empty vector (Fig. 2B).  Together these results indicate that the alleles of *Lr67res* that increase the sensitivity of yeast to NaCl also generate larger oocyte currents (Milne *et al.*, 2023) and confer pathogen resistance in wheat.

### An Lr67res ion-associated function can be phenocopied in other STP13s, and residues other than arginine can induce this phenotype

**Fig. 3.**
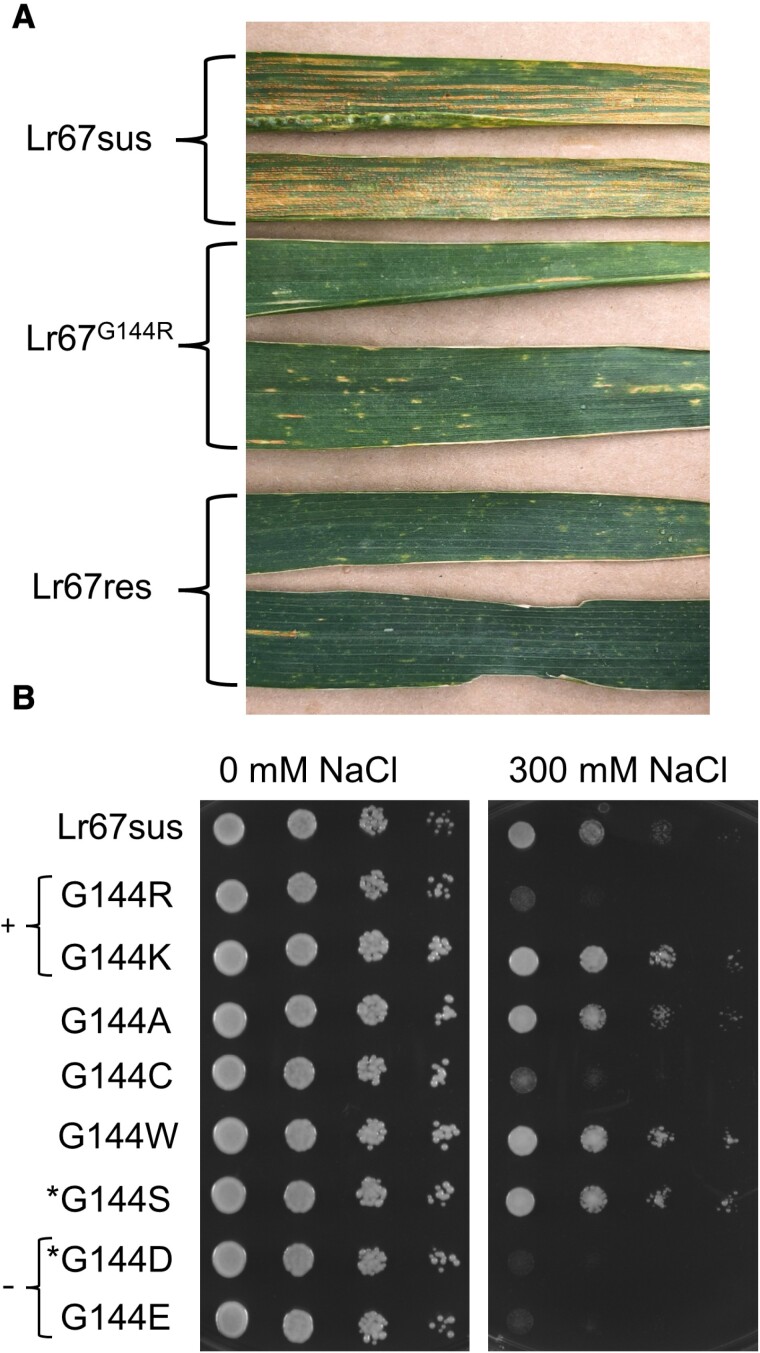
Disease resistance conferred by the G144R mutation in wheat and yeast NaCl sensitivity of *Lr67* site-directed mutants. (A) Flag leaves of field-grown cv. Avocet S wheat carrying *Lr67sus*, *Lr67*^*G144R*^, or *Lr67res*, infected with *P. striiformis* f. sp. *tritici*. (B) Decimal dilution series of EBY.VW4000 yeast, from an OD_600_ of 0.8, transformed with *Lr67sus*, or *Lr67sus* containing each of the site-directed mutants at position G144 grown on medium supplemented with NaCl. Images are representative of three biological replicates (independently transformed yeast colonies). Positively and negatively charged amino acids are labelled + and –, respectively, and changes achievable via chemical mutagenesis are marked with an asterisk *.

Since the *Lr67* alleles identified in wheat (*Lr67res* and *Lr67sus*) differ by two residues (G144R and V387L), we tested whether both changes are required to induce NaCl sensitivity in yeast. We found that the G144R mutation alone was sufficient to induce NaCl sensitivity in yeast but the single V387L mutation was not (Fig. 1A). This is consistent with our identification of another naturally occurring Lr67 variant in a wheat landrace (AUS4793) that confers resistance, despite the presence of only the G144R mutation. We introgressed this G144R allele from the donor landrace into the wheat cultivar Avocet S, which is highly susceptible to stripe rust, and confirmed that it confers the same partial resistance to wheat stripe rust as the *Lr67res* allele with both G144R and V387L mutations (Fig. 3A).

To further investigate the impact of amino acid substitutions other than arginine in place of the conserved glycine residue 144, synthetic variants were introduced into Lr67sus and tested in yeast for NaCl sensitivity. Mutating G144 to lysine (G144K), alanine (G144A), tryptophan (G144W), or serine (G144S) did not confer greater yeast NaCl sensitivity compared with the wild-type Lr67sus (Fig. 3B). Mutation to aspartic acid (G144D) or glutamic acid (G144E) conferred strong sensitivity resembling G144R, whereas mutation to cysteine (G144C) conferred an intermediate NaCl sensitivity in yeast. Two observations are made from these results: the change from glycine to arginine is not critical for NaCl sensitivity in yeast because it also occurs with G144D and G144E which are both negatively charged residues; and the properties of the amino acid at residue 144 that induce NaCl sensitivity are quite variable.

**Fig. 4.**
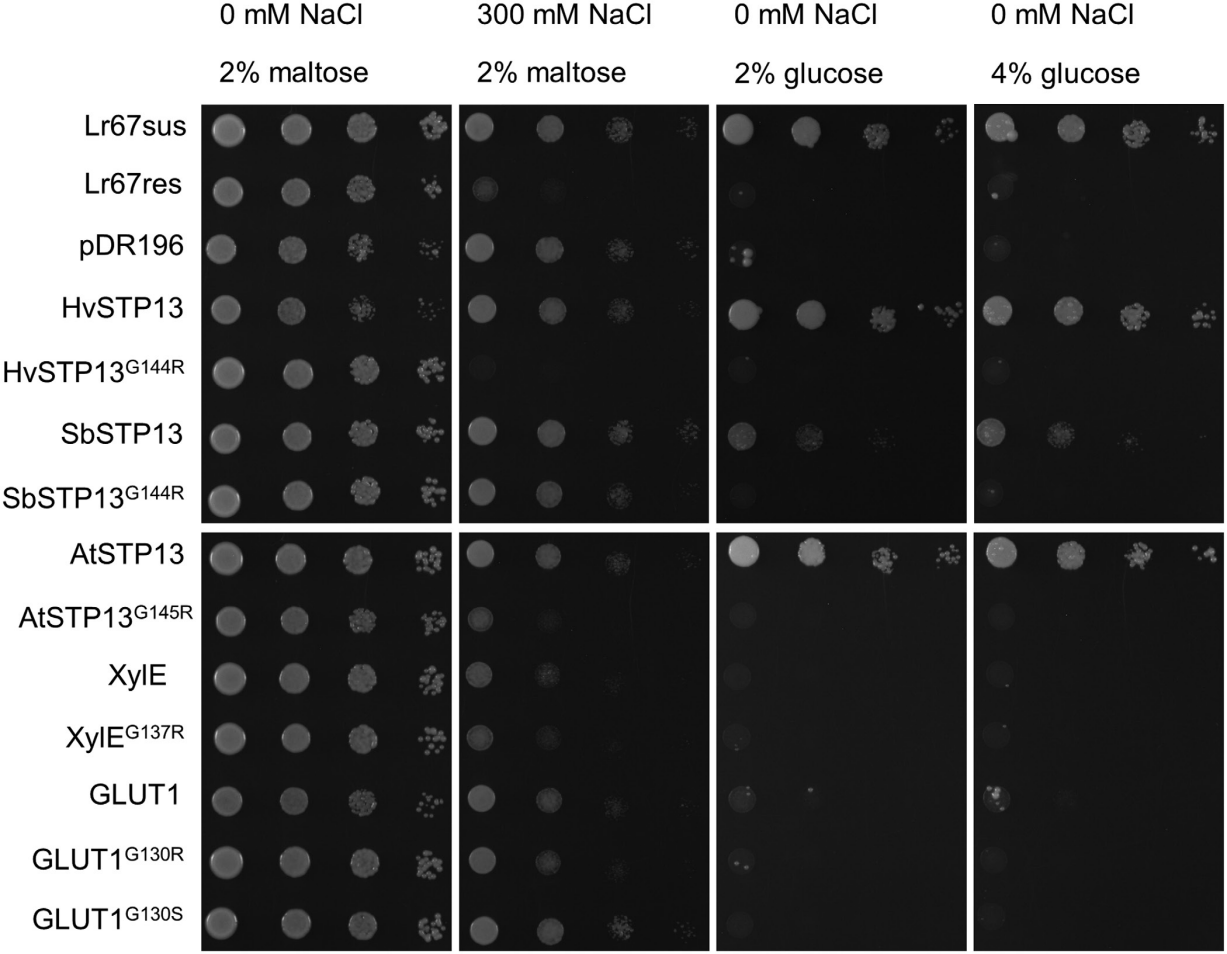
NaCl sensitivity and glucose uptake capability of yeast transformed with *Lr67*-related hexose transporters and site-directed mutants. Decimal dilution series of EBY.VW4000 yeast, from an OD_600_ of 0.8, transformed with the wheat *Lr67sus* and *Lr67res*, empty vector pDR196, barley *HvSTP13* and *HvSTP13*^*G144R*^, sorghum *SbSTP13* and *SbSTP13*^*G144R*^, Arabidopsis *AtSTP13* and *AtSTP13*^*G154R*^, *Escherichia coli XylE* and *XylE*^*G137R*^, and human *GLUT1*, *GLUT1*^*G130R*^, and *GLUT1*^*G130S*^. The medium was supplemented with NaCl as indicated, or maltose was substituted for glucose as the carbon source. Images are representative of three biological replicates (independently transformed yeast colonies).

Since the G144 equivalent residue is highly conserved in plant sugar transporters, it could represent a promising target for introducing disease resistance into other species, assuming that redundancy in the STP family can account for the loss of sugar transport function in STP13^G144R^. Conservation of residues was determined previously using the EvCouplings server (Milne *et al.*, 2019), and a multiple sequence alignment highlighting the G144 residue of STPs used in this study is shown in Supplementary Fig. S2. We introduced orthologous mutations into transporters from other species, expressed them in yeast, and tested whether they conferred sensitivity to NaCl. Introducing the single G144R mutation into the barley *HvSTP13* gene was sufficient to confer NaCl sensitivity to yeast. No sensitivity was evident when the V387L mutation was added alone, and no additional sensitivity was evident when it was combined with G144R in HvSTP13 (Fig. 4, cf. 0 mM NaCl with 300 mM NaCl; Supplementary Fig. S3A). Furthermore, the Arabidopsis AtSTP13^G145R^ protein and, to a lesser extent, the sorghum SbSTP13^G144R^ protein also exhibited increased NaCl sensitivity in yeast compared with their wild-type counterparts (Fig. 4). The *Medicago truncatula* MtSTP13.1 containing the G144R mutation, able to confer disease resistance (Gupta *et al*., 2021), also caused NaCl sensitivity in yeast compared with the MtSTP13.1 wild-type counterpart (Supplementary Fig. S3B). EBY.VW4000 yeast is deficient in glucose uptake and is unable to grow on medium containing glucose as the sole carbon source. Expression of *Lr67sus*, *HvSTP13*, *SbSTP13*, or *AtSTP13* in EBY.VW4000 could complement the growth phenotype, whereas introduction of the critical G144R (or equivalent change in those proteins) was unable to complement yeast growth on glucose medium, suggesting that they too lack the hexose transport function as demonstrated previously for Lr67res (Moore *et al*., 2015) and HvSTP13^G144R,V387L^ (Milne *et al.*, 2019) (Fig. 4, cf. 2% maltose with 2% and 4% glucose). By contrast, the structurally resolved bacterial xylose–proton symporter (XylE) (Sun *et al.*, 2012) and the human glucose facilitator (GLUT1) (Wang *et al.*, 2005; Deng *et al.*, 2014) were unable to complement yeast growth on glucose medium, and variants of each protein (XylE^G137R^, GLUT1^G130R^, and GLUT1^G130S^) did not enhance NaCl sensitivity (Fig. 4). Assuming all are correctly targeted to the yeast plasma membrane, these results suggest that generation of the NaCl-sensitive phenotype in yeast may only be possible in closely related plant transporters within the STP family.

### Extending observations from heterologous systems to plants

**Fig. 5.**
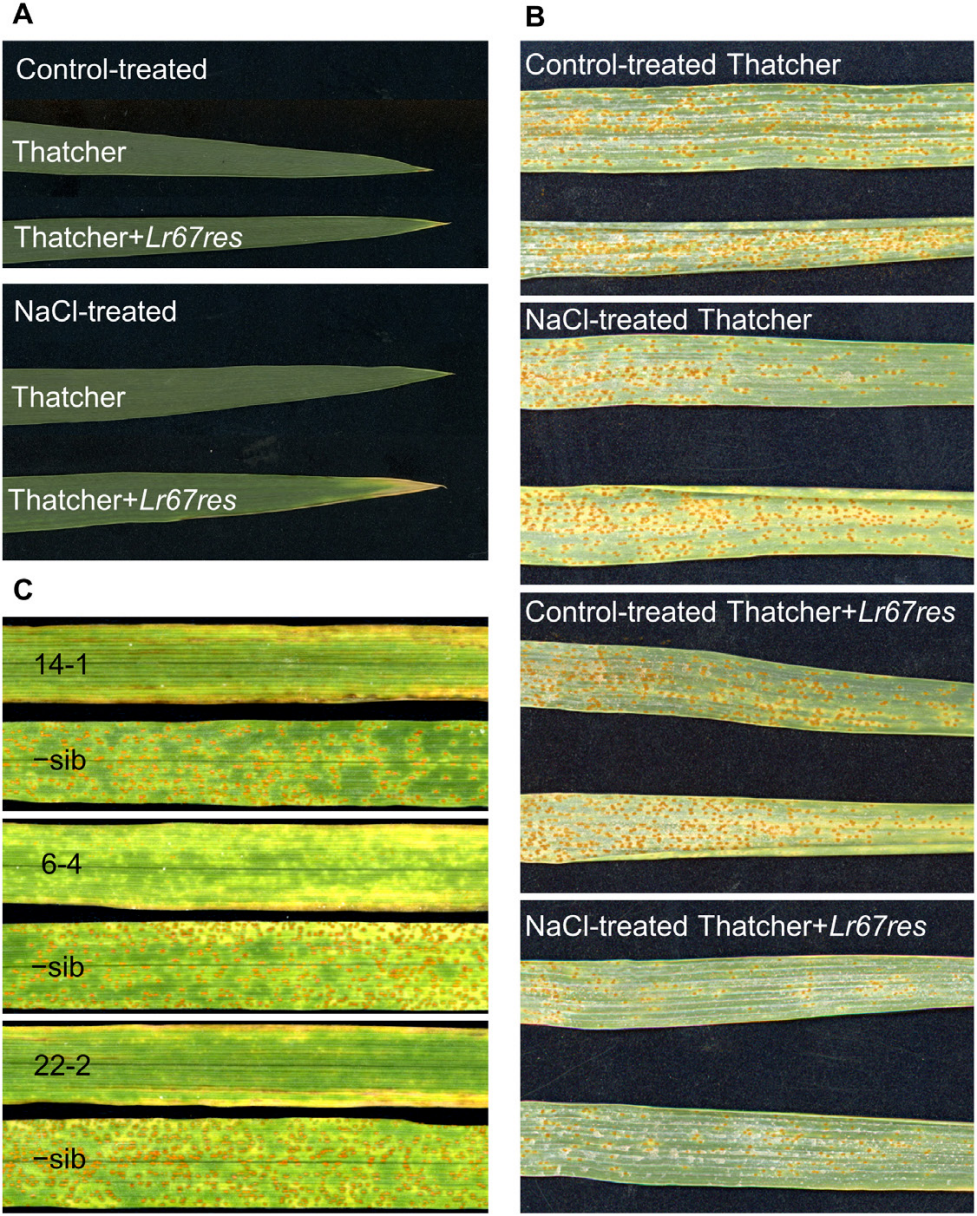
NaCl-induced wheat leaf tip necrosis and leaf rust resistance, along with rust resistance phenotype of barley transformed with *HvSTP13*^*G144R,V387L*^. (A) Flag leaves collected from the main tiller of representative wheat cv. Thatcher or Thatcher*+Lr67res* plants treated with half-strength Hoagland’s solution ±NaCl. A 25 mM NaCl treatment was applied at anthesis, followed by 50 mM NaCl treatment 6 d later, and leaves were sampled 5 d after the second treatment. Images are representative of 12 biological replicates and two independent experiments. Additional replicates are shown in Supplementary Fig. S4. (B) Flag leaves collected from the main tiller of two representative wheat cv. Thatcher or Thatcher*+Lr67res* plants treated as in (A), followed by *P. triticina* inoculation 4 d post-second salt treatment and photographed 9 d post-inoculation. Images are representative of eight biological replicates and two independent experiments. Additional replicates are shown in Supplementary Fig. S7. (C) Barley leaves from three independent transgenic events inoculated with *P. hordei*. Images represent leaves of barley plants carrying the *HvSTP13*^*G144R,V387L*^ transgene, or corresponding −sib lacking the transgene at 8 d post-inoculation.

To determine whether NaCl treatment could also induce distinct phenotypes in wheat plants expressing alleles of *Lr67*, as observed in yeast, near isogenic cv. Thatcher wheat lines carrying either the *Lr67sus* or *Lr67res* allele (Thatcher and Thatcher*+Lr67res*, respectively) were subjected to NaCl treatment during the adult stage of development. We established that Thatcher*+Lr67res* plants do not reliably display partial disease resistance or the leaf tip necrosis (Ltn) phenotype when grown in glasshouses or growth cabinets; rather, these phenotypes are only evident in field-grown adult plants, as opposed to seedlings with lower *Lr67* expression (Ramírez-González *et al.*, 2018; Milne *et al.*, 2019). However, we discovered that Ltn was induced on the flag leaves of cabinet-grown Thatcher*+Lr67res* adult plants once NaCl treatment commenced at anthesis—performed by watering pots once with a 25 mM NaCl solution, followed by a 50 mM NaCl solution 6 d apart. Induction of Ltn did not occur in Thatcher plants, or in plants of either genotype that did not receive NaCl treatment (Fig. 5A; Supplementary Fig. S4). Leaf tip necrosis was also evident on the penultimate leaves (Supplementary Fig. S4) and older leaves of NaCl-treated Thatcher*+Lr67res*, indicating that an Lr67res-dependent, ion-associated phenotype is transferrable to plants as well as yeast. Further, Ltn was induced by NaCl treatment in transgenic cv. Fielder and cv. Stewart wheat (representing hexaploid and tetraploid genotypes, respectively; Supplementary Fig. S5). Negative segregants (−sibs) of each cultivar showed no difference in Ltn phenotype ±NaCl treatment, whereas greater Ltn symptoms were observed in two independent transgenic events of each cultivar positive for *Lr67res* (Supplementary Fig. S5). There was a higher background Ltn in control-treated *Lr67res* transgenics as compared with Thatcher*+Lr67res* that may be due to either higher *Lr67res* expression or possibly genetic background effects.

Since Lr67res rust disease resistance is generally associated with the Ltn phenotype, flag leaves of Thatcher and Thatcher*+Lr67res* wheat lines grown in cabinets were inoculated with *P. triticina* 4 d after the conclusion of NaCl treatment to determine whether disease resistance is also triggered by the NaCl. Reduced fungal colonization was observed in NaCl-treated Thatcher*+Lr67res* plants in comparison with control-treated Thatcher*+Lr67res*, whereas fungal colonization of control and NaCl-treated Thatcher plants was equal (Fig. 5B; Supplementary Fig. S6). Microscopic analysis of WGA–FITC-stained flag leaves revealed a reduction in size and number of *P. triticina* infection sites of NaCl-treated Thatcher*+Lr67res* plants at 5 dpi (Supplementary Fig. S6). After a longer time period post-NaCl treatments (13 d as opposed to 6 d in Fig. 5A), Ltn symptoms were more evident in NaCl-treated Thatcher*+Lr67res* flag leaves than in plants that did not receive the NaCl treatment or in Thatcher plants (Supplementary Fig. S7).

**Fig. 6.**
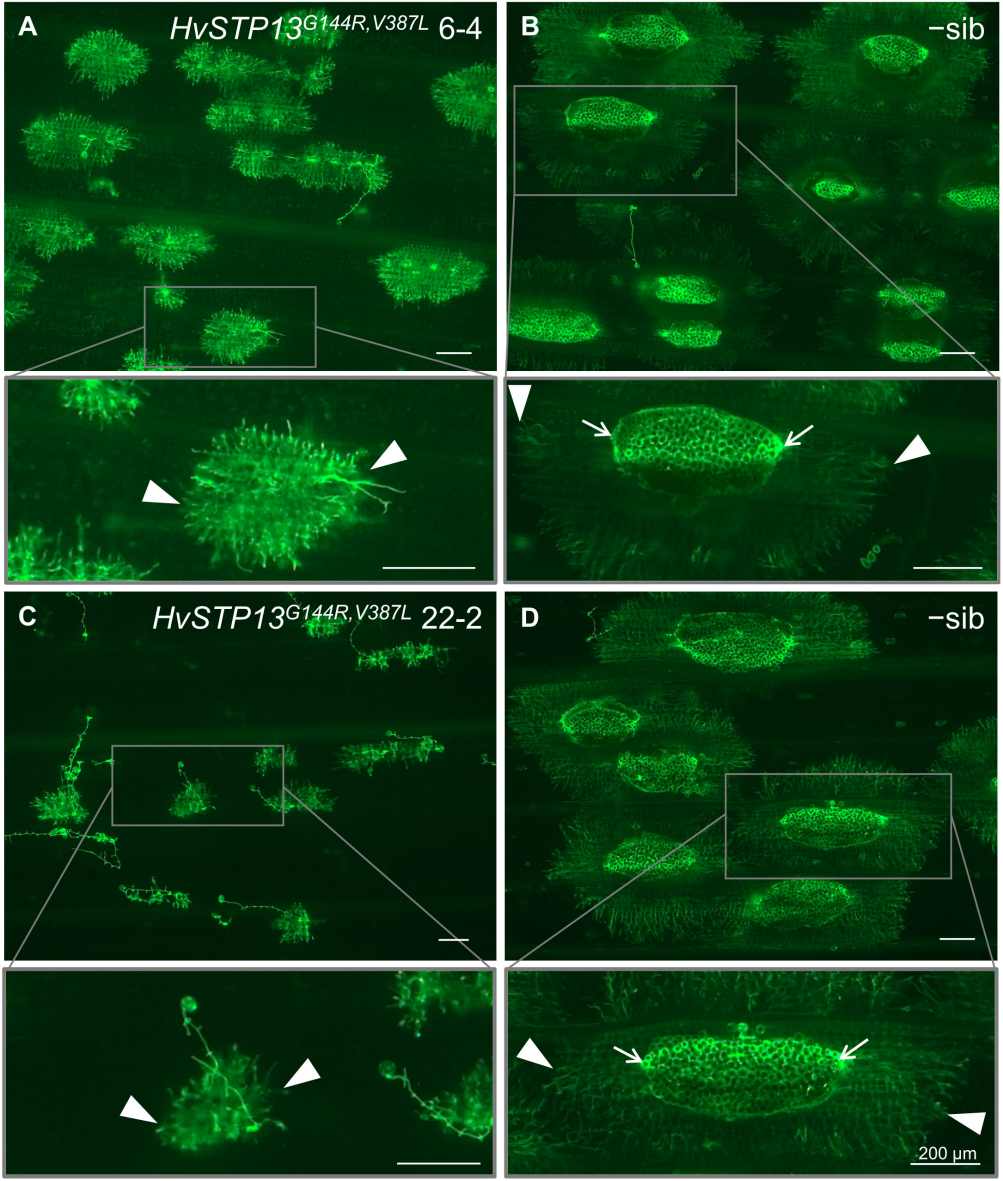
Fluorescence micrographs of WGA–FITC-stained barley cv. Golden Promise leaves, highlighting the absence of sporulation in transgenic events at 8 d post-inoculation with *P. hordei*. Representative images for plants exhibiting (A) intermediate and (C) strong resistance phenotypes harvested from transgenic lines expressing *HvSTP13*^*G144R,V387L*^ where hyphal development was present but no sporulation. (B, D) Corresponding susceptible segregants, −sibs, lacking the *HvSTP13*^*G144R,V387L*^ transgene, exhibiting extensive fungal colonization and sporulation. Insets below (A–D) depict magnified images of the outlined infection site. Arrowheads indicate hyphae at the periphery of the infection site, whilst arrows indicate the presence of sporulation. Scale bars=200 µm.

To further translate the observations made with the synthetic *HvSTP13* variants in yeast to whole plants, a construct was prepared containing the full genomic sequence of the modified *HvSTP13* with both mutations (*HvSTP13*^*G144R,V387L*^) along with 1503 bp of its native promoter and a 1660 bp 3'-UTR. The construct was transformed into barley cv. Golden Promise, and T_1_ plants underwent rust disease screening. Multiple independent transgenic events containing the *HvSTP13*^*G144R,V387L*^ transgene exhibited partial disease resistance to barley leaf rust when inoculated with *P. hordei*, with three independent events presented, whereas their non-transgenic sibling lines (−sibs) remained susceptible (Fig. 5C). The severity of disease symptoms was also assessed microscopically because the presence or absence of sporulation in the infected leaves is a reliable indicator of greater or lesser disease progression, respectively. Microscopic examination of inoculated leaves was compared in the 22-2 and 6-1 events along with their −sib controls, and the extent of sporulation was recorded. This microscopic analysis distinguished between strong and intermediate resistance phenotypes at 8 dpi (Fig. 6) which became visible macroscopically at 13 dpi (Supplementary Fig. S8). This is significant because strong resistance conferred by Lr67res is not typically observed in wheat. Hyphal development and sporulation were evident in −sibs lacking *HvSTP13*^*G144R,V387L*^ (Fig. 6B, D). Hyphal development in the absence of sporulation was observed in transgenic event 6-4 showing intermediate resistance (Fig. 6C), whereas only minimal hyphal development in the absence of sporulation was evident in the transgenic event 22-2, showing strong resistance. Interestingly, the severe pleiotropic effects previously observed in barley when the wheat *Lr67res* gene was stably expressed under its native promoter (Milne *et al.*, 2019) were reduced by expressing *HvSTP13*^*G144R,V387L*^ under its own promoter. This was evident in the reduction of early senescence, a more normal growth habit, and fully filled grains. Collectively, these results demonstrate that site-directed mutations to *Lr67* genes from other crop species can be easily tested for their potential to confer partial disease resistance *in planta* by first expressing them in yeast and screening for increased sensitivity to NaCl.

## Discussion

While the single amino acid change G144R in Lr67 removes its capacity to transport sugar and allows it to confer disease resistance, the resistance mechanism remains unclear. We have established that the heightened ion transport functions generated by *Lr67res* expression in *Xenopus* oocytes have now been extended to other biological systems. These include the increased sensitivity of yeast to NaCl and the NaCl-induced leaf tip necrosis coupled with partial disease resistance in adult wheat plants. We previously attributed the Lr67res-dependent ion activity in oocytes to enhanced permeability to Cl^−^ and other anions (Milne *et al.*, 2023). We predict that the NaCl screen we developed in yeast works as follows: (i) expression of *Lr67res* (and similar alleles) increases permeability to Cl^−^; (ii) at high external NaCl (or LiCl) concentrations, the influx of Cl^−^ into the cells is increased; (iii) this accumulation of anions in the cells is balanced by the accumulation of Na^+^, the only major cation present, to maintain electroneutrality; and (iv) the accumulation of Na^+^ becomes toxic and the cells die. This yeast screen enables the rapid detection of the novel ion fluxes associated with Lr67res function without specialized electrophysiology equipment. Indeed, it can be a proxy screen for disease resistance. Synthetic variants from the *STP13* gene family can first be tested for sensitivity to NaCl prior to proceeding to the much greater investment required for the development of transgenic plants.

### Yeast represents a useful system to test and characterize novel resistance genes

A curious observation made when *Lr67res* was expressed in *Xenopus* oocytes was the increase in cytosolic Na^+^ content compared with *Lr67sus* and control oocytes (Milne *et al.*, 2023). We now interpret this as enhanced Cl^−^ uptake via Lr67res as being balanced by the accumulation of Na^+^ to maintain electroneutrality. This conclusion led us to compare yeast growth on media supplemented with various salts (including NaCl; Fig. 1A) and, although we have not quantified internal Na^+^ in yeast, we presume Na^+^ accumulation is the reason for the Lr67res-induced sensitivity on high NaCl media. The finding that similar high concentrations of KCl are not toxic to yeast, in the same way that NaCl is, supports this conclusion, as do observations with yeast harbouring a known chloride channel, TmClC-0, despite the lower degree of NaCl sensitivity. Given our previous conclusion that anions contribute to the Lr67res-dependent currents detected in oocytes more than cations (Milne *et al.*, 2023), the TmClC-0 anion channel was also tested in yeast under high NaCl. This transporter also conferred an NaCl-sensitive phenotype, albeit less severe than that conferred by Lr67res (Fig. 1A). This may potentially be due to variations in the level of expression between *Lr67res* and *TmClC-0* in yeast. Additionally, sensitivity to KI was observed in Lr67res yeast, indicating elevated uptake of I^−^, known to be toxic to yeast (Greaves *et al.*, 1928). This is in support of our hypothesis that the transport of chloride, and/or other anions, is key to the Lr67res-mediated disease resistance mechanism *in planta*.

It remains unclear exactly what factors determine whether NaCl sensitivity can be introduced into other transporters. The synthetic barley, Arabidopsis, and sorghum STP13^G144R^ (or equivalent) variants that conferred NaCl sensitivity to yeast (Fig. 4) were between 98.8% and 81.4% identical to Lr67 at the amino acid level, whereas the bacterial XylE^G137R^ and human GLUT1^G130R^ which did not confer yeast NaCl sensitivity shared far lower peptide sequence identity to Lr67 (~27% identical). Despite their low sequence identity, the known and predicted structures of these transport proteins are very similar, which suggests that particular residues or regions of the protein may be a factor. Specifically, an extracellular disulfide bond in the lid domain between Cys77 and Cys449 identified in the AtSTP10 crystal structure (Paulsen *et al.*, 2019) may be required for the Lr67res function. The equivalent C75Y LOR mutation of Lr67res abolished resistance in wheat (Spielmeyer *et al.*, 2013; Moore *et al.*, 2015) and reduced NaCl sensitivity in yeast (Fig. 2A). Moreover, the corresponding amino acid is not conserved in XylE or GLUT1, which may explain why XylE^G137R^ and GLUT1^G130R^ did not phenocopy the yeast NaCl sensitivity.

In terms of understanding the function associated with Lr67res, it is puzzling that amino acid changes other than G144R, yet with similar properties to arginine, do not necessarily induce an Lr67res-like NaCl sensitivity phenotype in yeast (Fig. 3B). For example, the change to lysine (G144K), which is positively charged like arginine, did not induce sensitivity, whereas mutations to glutamate and aspartate (G144D and G144E), both of which are negatively charged, did induce NaCl sensitivity in yeast. It is possible that negatively charged residues (G144D and G144E) may allow passage of different ions in contrast to G144R. Amino acid size may not be a major factor either given that the large uncharged polar side chain amino acid tryptophan (G144W) was unable to induce NaCl sensitivity in yeast. Specific interactions may occur between the amino acid at position 144 and neighbouring amino acids that contribute to Lr67res function. It may also be possible that certain mutations could impact correct protein folding and/or targeting to the plasma membrane, resulting in a lack of yeast NaCl sensitivity. Coincidentally, the point mutation G144D is likely to be achievable via chemical mutagenesis (see Milne *et al.*, 2023 for STP13 sequence analysis) and, since this change induces NaCl sensitivity in yeast (Fig. 3B), it would be worthwhile to screen mutant populations for an equivalent mutation in target species to test for Lr67res-like disease resistance.

### Bridging the gap between heterologous expression systems and plants

In the context of developing new sources of disease resistance, our finding that an Lr67res-like NaCl sensitivity in yeast can be transferred to related transporters is important. Results from this yeast screen predicted that the single G144R mutation in wheat and barley STP13s should confer disease resistance *in planta*. Indeed, this was shown to be the case in wheat (Fig. 3A) and barley (Skoppek *et al*., 2022). Like wheat, the barley HvSTP13^G144R,V387L^ also confers a disease resistance phenotype (Figs 5B, 6). Our yeast observations also extend to the dicot transporter from *M. truncatula*, MtSTP13.1, where the MtSTP13.1^G144R^ variant conferred yeast NaCl sensitivity (Supplementary Fig. S3) and powdery mildew resistance when transiently expressed in pea leaves (Gupta *et al*., 2021). The veracity of the yeast screen is further supported by the LOR mutants previously identified *in planta* which lost disease resistance and the Ltn phenotype (Spielmeyer *et al.*, 2013; Moore *et al.*, 2015). These LOR mutants showed reduced sensitivity to NaCl in yeast and reduced current magnitudes in oocytes. The incomplete loss of yeast NaCl sensitivity (Fig. 2A) and the residual oocyte currents for one of the LOR mutants (Lr67res^G208D^) (Milne *et al.*, 2023) could be interpreted to mean that a threshold of anion transport needs to be met for resistance. Leading on from these observations, the NaCl-dependent induction of Ltn in wheat plants (Fig. 5A) provides further evidence that ion transport and/or ion homeostasis may also be a key factor for this phenotype in plants.

Observations confirm that both HvSTP13^G144R^ (Skoppek *et al.*, 2022) and HvSTP13^G144R,V387L^ (Figs 5B, 6) stably expressed in barley cv. Golden Promise are able to confer resistance to *P. hordei* and that the V387L change does not appear to confer any greater resistance. In addition, we observed much improved growth, reduced early senescence, and higher grain yields in the transgenic barley expressing HvSTP13^G144R,V387L^ compared with the same cultivar transformed with the wheat *Lr67res* gene (Milne *et al.*, 2019). Limited evidence exists on whether loss of the normal STP13 sugar transport function reduces yield or compromises plant growth vigour. In wheat, partial deletions of the D genome STP13 homeologue would be difficult to interpret since functional A and B genome STP13 homeologues were still present (Moore *et al*., 2015). In Arabidopsis, *stp13* T-DNA insertional mutants were phenotypically normal under standard growth conditions (Nørholm *et al.*, 2006) and under various seedling growth treatments (Schofield *et al.*, 2009). Conversely, RNAi knockdown of the *STP13* orthologue in tomato (*LeHT2*) reduced fruit hexose accumulation despite otherwise normal plant growth (McCurdy *et al.*, 2010). Assessment of whether the G144R mutation, rendering STP13 incapable of sugar transport, causes negative implications in terms of sugar transport may need to be assessed on a species by species basis.

In the context of crop breeding and whether modified *STP13* resistance alleles have the potential to be used commercially in crops outside of wheat, using the recipient species’ native *STP13* gene with G144R incorporated seems thus far to be a more logical strategy than using the wheat *Lr67res* gene itself. Given this, it would be interesting to compare the performance of HvSTP13^G144R^ gene-edited barley alongside HvSTP13^G144R^ transgenic plants. Should editing the G144R change not be feasible, other transgenic strategies to minimize pleiotropic effects during plant development may be to utilize developmentally dependent promoters that result in expression only in adult plants [mimicking when adult plant resistance (APR) would typically be observed], or possibly pathogen-responsive promoters to drive expression of resistance alleles when pathogens are encountered would be more useful. The latter strategy has been demonstrated for *Lr34res* in barley (Boni *et al.*, 2018) which minimized pleiotropic effects whilst still conferring resistance.

### The connection between Lr67res-dependent plant immunity and anion fluxes

Based on the data presented, we propose that a change in ion fluxes is likely to be a biologically relevant factor for *in planta* multipathogen disease resistance for two reasons. Firstly, the yeast ion sensitivity phenotypes (or lack of sensitivity in the case of LOR mutants) corresponded in disease resistance phenotypes when tested in both wheat and barley plants. Secondly, the induction of Ltn and partial rust resistance by NaCl treatment, in growth conditions not otherwise conducive to the development of both Ltn and partial disease resistance, suggests that both are linked with the Lr67res-dependent anion transport activity (Fig. 5A, B). In addition to *Lr67*, the Ltn phenotype is also known to be linked with disease resistance phenotypes for the other multipathogen resistance genes *Lr34* and *Lr46* (Singh *et al.*, 1998; Krattinger *et al.*, 2009; Moore *et al.*, 2015). Whether wheat carrying *Lr34* or *Lr46* also shows an induction of Ltn under NaCl treatments may shed light on involvement in common pathways.

Anion fluxes, and in particular those of chloride, have been implicated in disease resistance and immune signalling (Liu *et al.*, 2019), with evidence accumulating that chloride channels and transporters can be both positive (Han *et al.*, 2019) and negative (Guo *et al.*, 2014) regulators of pathogen-associated molecular pattern (PAMP)- and effector-triggered immunity. Additionally, abscisic acid (ABA), which is the transport substrate of the other characterized multipathogen resistance protein, Lr34 (Krattinger *et al.*, 2019), triggers channel-mediated Cl^−^ fluxes (Roelfsema *et al.*, 2004), and induces leaf senescence (Mao *et al.*, 2017). Occurrence of Ltn in plants carrying an *APR* gene in the absence of pathogen infection indicates that physiological changes leading to Ltn and resistance are not necessarily reliant on pathogen induction. Instead, we propose that Lr67res-associated ion fluxes *in planta* underpin both multipathogen resistance and the observed Ltn induction, and future experiments will work towards characterizing whether these phenotypic responses are due to localized perturbations in ion distribution, or more systemic ion-induced abiotic stress signalling. In terms of general functionality of multipathogen resistance proteins in wheat, Lr67 appears to possess a molecular function distinct from that of Lr34, since Lr34 did not induce the same sensitivity to NaCl (Supplementary Fig. S3B), nor did it induce large currents in oocytes (Milne et al., 2023), which reconciles with involvement of Lr34 in a distinct transport process from Lr67 (ABA transport) (Krattinger *et al.*, 2019). In contrast to the classical immune response involving gene-for-gene recognition which has recently been linked to calcium ion permeability culminating in the hypersensitive response and cell death (Bi *et al.*, 2021; Jacob *et al.*, 2021), the partial multipathogen resistance and durability of both Lr67res and Lr34res could instead rely on changes to ion permeability of the host cell’s plasma membrane, rendering the host tissues less conducive to pathogen growth and virulence, in the absence of the hypersensitive response, through an as yet unidentified mechanism.

In this study, an ion-associated function has been demonstrated to occur in yeast as NaCl sensitivity and *in planta* by NaCl induction of leaf tip necrosis, concurrent with partial disease resistance. The application of NaCl as a form of abiotic stress poses an interesting scenario where crosstalk between abiotic and biotic stress may take place in the pathway in which Lr67res functions. The lack of specialized equipment required to detect phenotypes associated with Lr67res makes assays used here useful tools for developing and characterizing novel sources of disease resistance when modifying STP13 in other plant species.

## Supplementary data

The following supplementary data are available at *JXB* online.

Fig. S1. Characterizing the Lr67res function in yeast.

Fig. S2. Multiple sequence alignment highlighting conservation of the G144 residue of sequences used in this study.

Fig. S3. The effect of *HvSTP13* site-directed mutants and G144R mutation of MtSTP13.1 on NaCl sensitivity in yeast.

Fig. S4. NaCl-induced leaf tip necrosis in Thatcher and Thatcher+*Lr67res* wheat.

Fig. S5. NaCl-induced leaf tip necrosis in Fielder and Stewart wheat backgrounds.

Fig. S6. Quantification and microscopic analysis of *P. triticina* infection in control and NaCl-treated wheat.

Fig. S7. NaCl-treated wheat infected with *P. triticina*.

Fig. S8. *P. hordei-i*nfected transgenic barley leaves at 13 dpi.

Table S1. Primers used in this study.

Table S2. Amplified and synthesized fragments and constructs used in this study.

erae164_suppl_Supplementary_Figure_S1_S8_Tables_S1_S2

## Data Availability

The data that support the findings of this study are available within the paper and its supplementary data.
