# Supplementary material for: Dissecting the causal polymorphism of the Lr67res multipathogen resistance gene
Source: J Exp Bot. 2024 May 15;75(13):3877–90. doi: 10.1093/jxb/erae164 (PMC11233415; doi:10.1093/jxb/erae164)
Supplement: erae164_suppl_Supplementary_Figure_S1_S8_Tables_S1_S2 [file erae164_suppl_supplementary_figure_s1_s8_tables_s1_s2.pdf]

Dissecting the causal polymorphism of the *Lr67res* multipathogen resistance gene

Ricky J Milne[1*], Katherine E Dibley[1], Jayakumar Bose[2,3], Adnan Riaz[1,4], Jianping Zhang[1,5], Wendelin Schnippenkoetter[1], Anthony R Ashton[1], Peter R Ryan[1], Stephen D Tyerman[2], Evans S Lagudah[1*]

SUPPLEMENTARY DATA

Supplementary Fig. S1. Characterising the Lr67res function in yeast.

Supplementary Fig. S2: Multiple sequence alignment highlighting conservation of G144 residue of sequences used in this study

Supplementary Fig. S3. The effect of *HvSTP13* site-directed mutants and G144R mutation of MtSTP13.1 on NaCl sensitivity in yeast.

Supplementary Fig. S4. NaCl-induced leaf tip necrosis in Thatcher and Thatcher+*Lr67res* wheat.

Supplementary Fig. S5. NaCl-induced leaf tip necrosis in Fielder and Stewart wheat backgrounds.

Supplementary Fig. S6. Quantification and microscopic analysis of *P. triticina* infection in control and NaCl treated wheat.

Supplementary Fig. S7. NaCl-treated wheat infected with *P. triticina*.

Supplementary Fig. S8: *P. hordei* Infected transgenic barley leaves at 13 dpi

Supplementary Table S1. Primers used in this study.

Supplementary Table S2. Amplified and synthesised fragments and constructs used in this study.

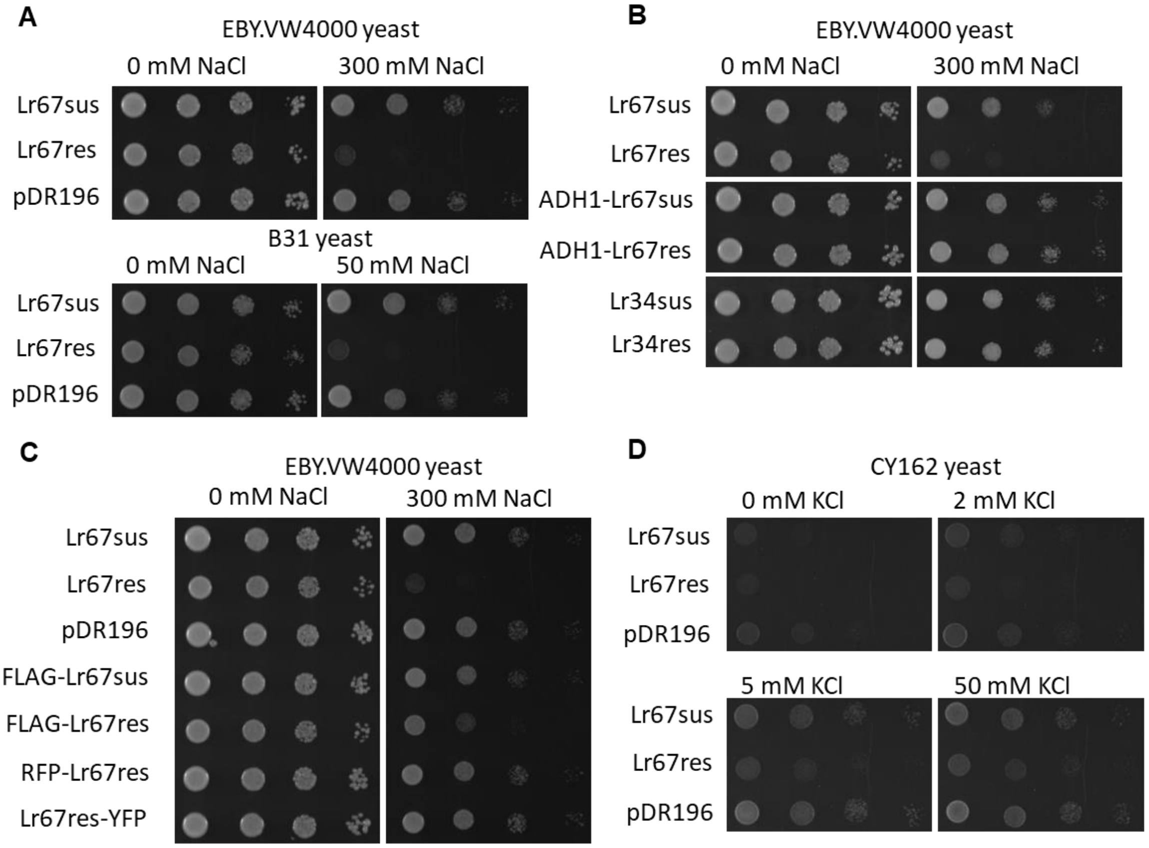

Supplementary Fig. S1. Characterising the Lr67res gain-of-function in yeast. Decimal dilution series of **A**, EBY.VW4000 hexose uptake deficient and B31 Na^+ efflux deficient yeast strains transformed with *Lr67sus*, *Lr67res* or pDR196 empty vector; **B**, EBY.VW4000 yeast expressing *Lr67sus* and *Lr67res* driven by the strong *PMA1* promoter and the weaker *ADH1* promoter; *Lr34sus* and *Lr34res* alleles driven by the strong *PMA1* promoter; **C**, EBY.VW4000 yeast transformed with *Lr67* alleles, the pDR196 empty vector, N-terminal *FLAG-Lr67* fusions, N-terminal *RFP-Lr67res* fusion and C-terminal *Lr67res-YFP* fusion; **D**, CY162 potassium uptake deficient yeast transformed with *Lr67sus*, *Lr67res* or pDR196 empty vector grown on low K^+ AP media, supplemented with KCl as indicated. Lr67res yeast were not capable of rescuing the K^+ uptake deficient phenotype to a greater extent than Lr67sus and pDR196, indicating K^+ transport is unlikely. Images are representative of three biological replicates (independently transformed colonies).

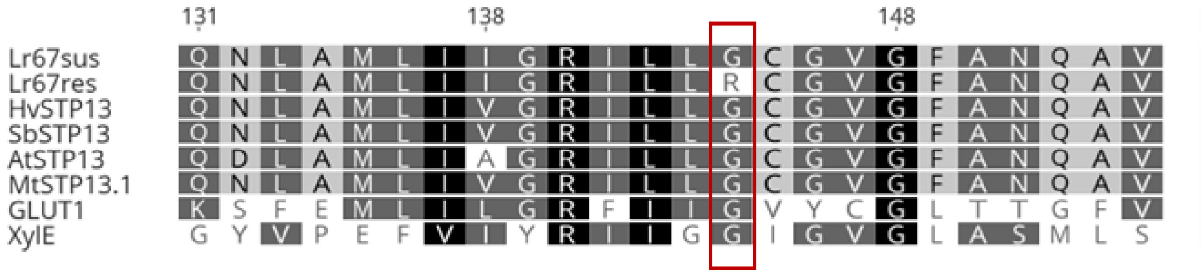

Supplementary Fig. S2: Multiple sequence alignment highlighting conservation of G144 residue of sequences used in this study. Amino acid sequence from region of STP13 (or most closely related transporter in the case of GLUT1 and XylE) containing G144 from *Triticum aestivum*, Lr67sus and Lr67res; *Hordeum vulgare*, HvSTP13; *Sorghum bicolor*, SbSTP13; *Arabidopsis thaliana*, AtSTP13; *Medicago truncatula*, MtSTP13.1; *Homo sapiens*, GLUT1; E. coli, XylE. The G144 residue is highlighted by a red box and is only an arginine (R) in Lr67res.

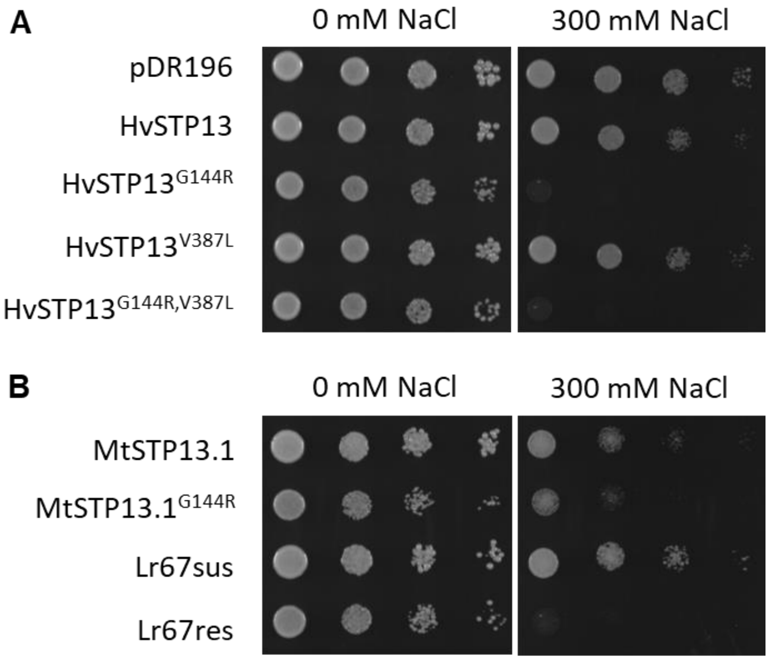

Supplementary Fig. S3. The effect of *HvSTP13* site-directed mutants and G144R mutation of MtSTP13.1 on NaCl sensitivity in yeast. Decimal dilution series of EBY.VW4000 yeast, from an OD_{600} of 0.8, transformed with **A**, pDR196 empty vector, *HvSTP13* and site-directed mutants *HvSTP13*^{G144R}, *HvSTP13*^{V387L}, *HvSTP13*^{G144R,V387L} or **B**, *MtSTP13.1* and *MtSTP13.1*^{G144R} grown on media supplemented with NaCl as indicated. Images are representative of three biological replicates (independently transformed colonies).

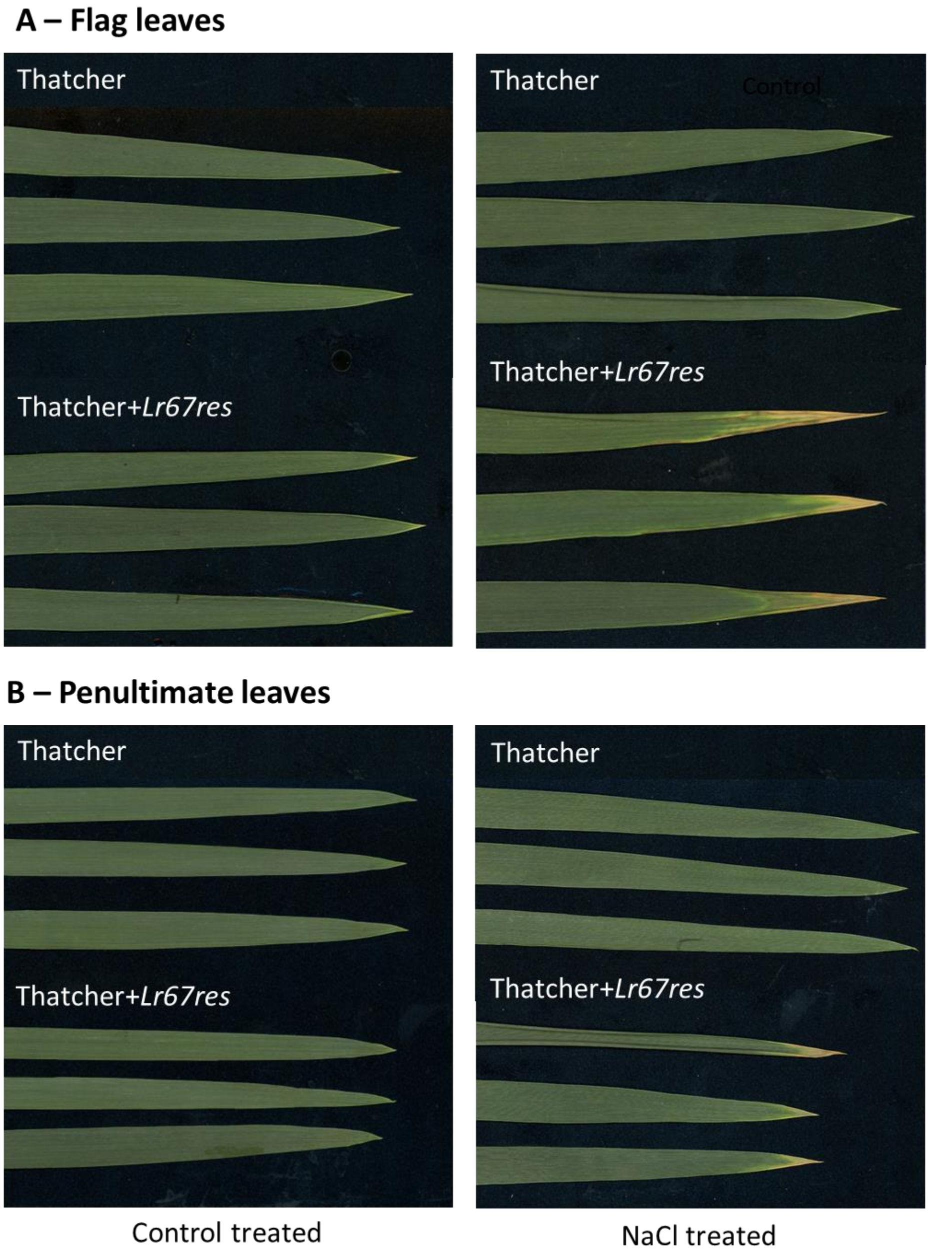

Supplementary Fig. S4. NaCl-induced leaf tip necrosis in Thatcher and Thatcher+*Lr67res* wheat. Three biological replicates are depicted to support Fig. 4A. **A,** Flag leaves or **B**, penultimate leaves collected from the main tiller of representative wheat cv. Thatcher or Thatcher+*Lr67res* plants treated with half-strength Hoaglands solution ± NaCl. A 25 mM NaCl treatment was applied at anthesis, followed by 50 mM NaCl treatment 6 days later and leaves were sampled 5 days after the second treatment. Images are representative of 12 biological replicates and two independent experiments.

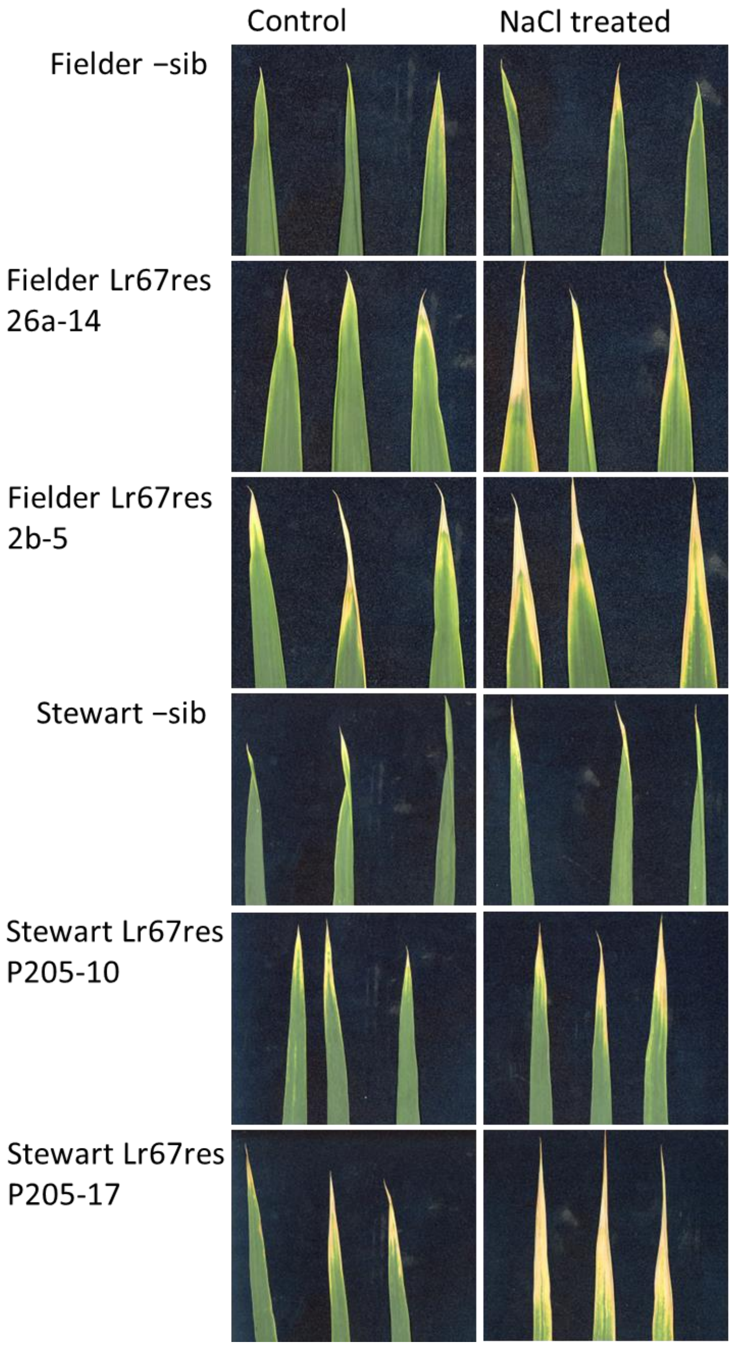

Supplementary Fig. S5. NaCl-induced leaf tip necrosis in Fielder and Stewart wheat backgrounds. Flag leaves collected from the main tiller of representative wheat cv. Fielder or cv. Stewart null segregants or independent transgenic events carrying *Lr67res*. Plants underwent treatment with half-strength Hoaglands solution ± NaCl. A 25 mM NaCl treatment was applied at anthesis, followed by 50 mM NaCl treatment 6 days later and leaves were sampled 5 days after the second treatment. Images are representative of observations made on 6 biological replicates.

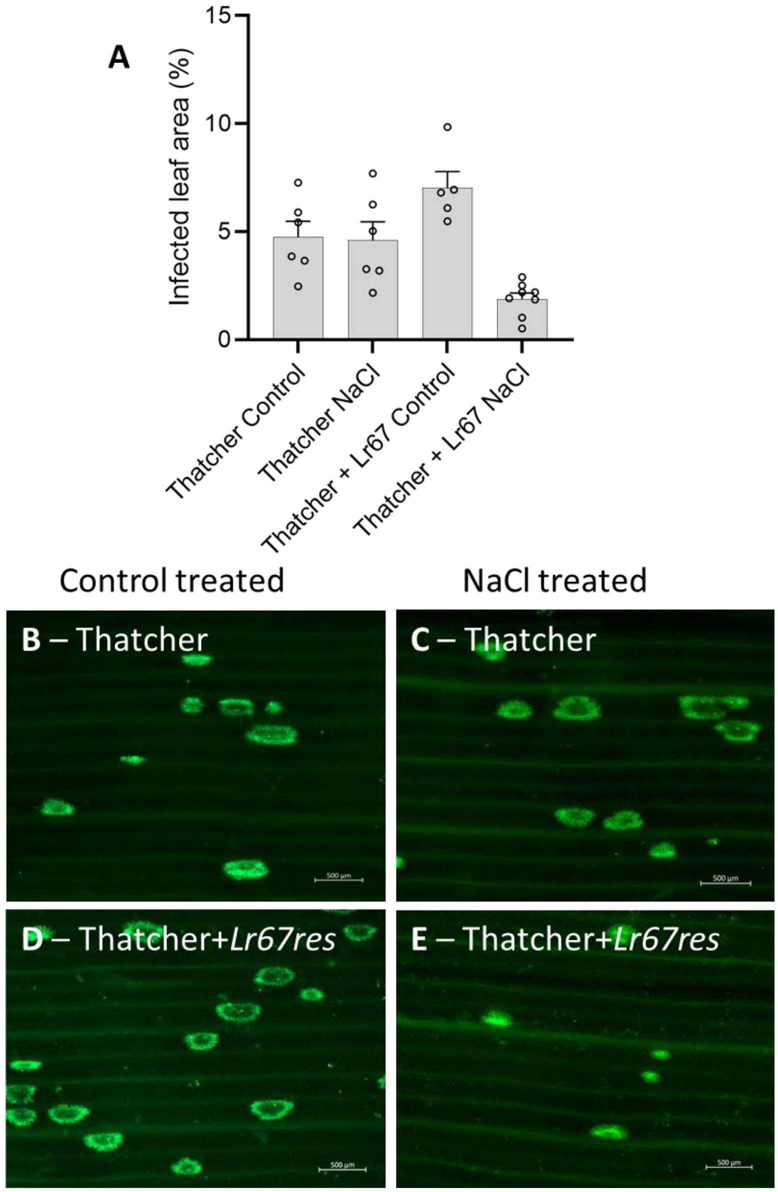

Supplementary Fig. S6. Quantification and microscopic analysis of *P. triticina* infection in control and NaCl treated wheat. A, Rust disease infected area of leaves presented in Fig. S5 was quantified using ImageJ software and expressed as a percentage of total leaf area. Columns with vertical bars represent the mean ± SE of 6-8 biological replicates (flag leaves from individual plants). **B-E**, microscopic representation of chitin stained with WGA-FITC illustrating rust development on flag leaves at 5 dpi in control treated (B,D) and NaCl treated (C,E) Thatcher and Thatcher+*Lr67res* plants respectively. Images are representative of samples taken from 4 biological replicates (individual plants).

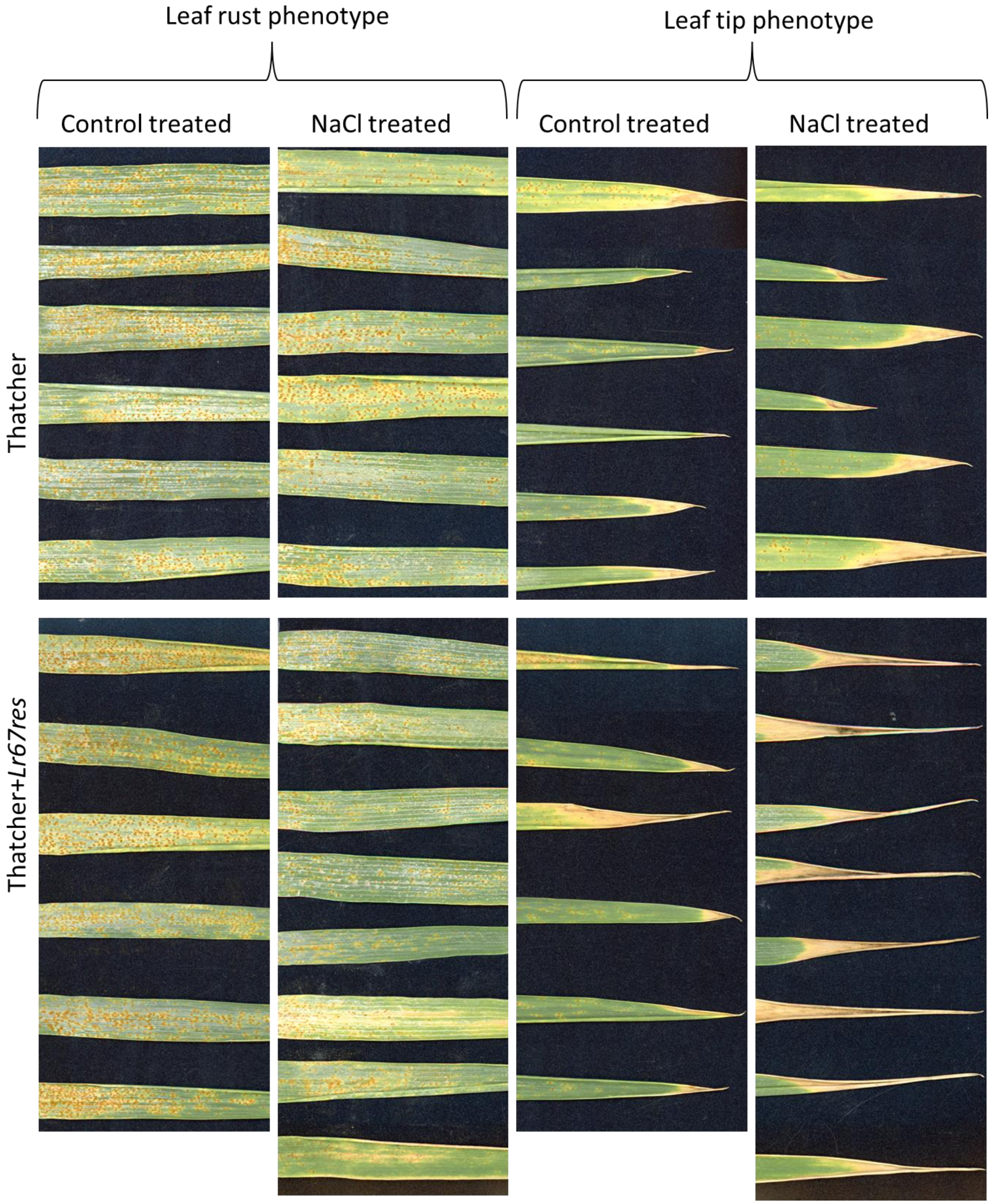

Supplementary Fig. S7. NaCl-treated wheat infected with *P. triticina*. Flag leaves collected from the main tiller of representative wheat cv. Thatcher or Thatcher + Lr67 plants 13 days post salt treatment and 9 days post inoculation with wheat leaf rust. n=6-8 biological replicates (each leaf represents a biological replicate).

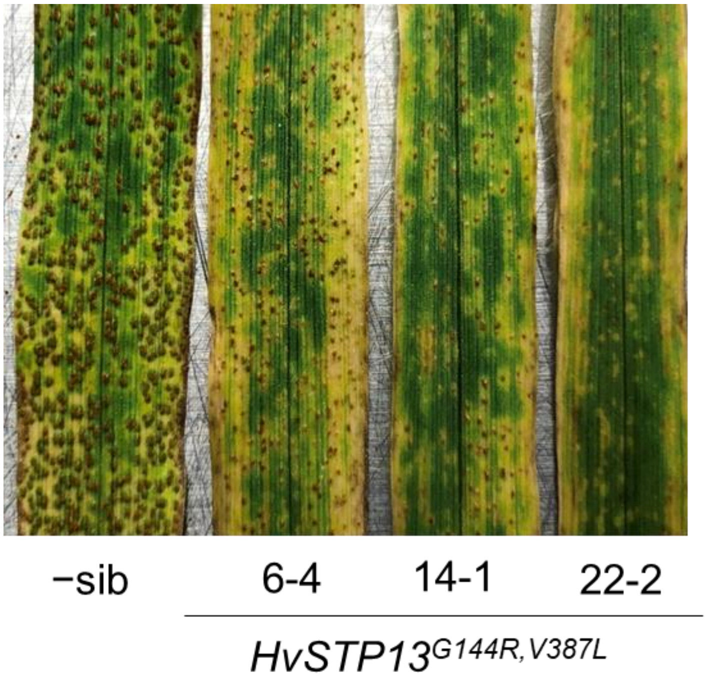

Supplementary Fig. S8: *P. hordei* Infected transgenic barley leaves at 13 dpi. −sib and independent *HvSTP13*^{G144R,V387L} transgenic events exhibiting various levels of sporulation.

Supplementary Table S1. Primers used in this study.

**Primer**	**Sequence (5'-3')**
Site-directed mutagenesis	
Lr67 G144A FP	GGATCCTGCTTGCTTGCGGCGTCGG
Lr67 G144A RP	CCGACGCCGCAAGCAAGCAGGATCC
Lr67 G144C FP	CAGGATCCTGCTTTGTTGCGGCGTCGG
Lr67 G144C RP	CCGACGCCGCAACAAAGCAGGATCCTG
Lr67 G144D FP	AGGATCCTGCTTGATTGCGGCGTCGGC
Lr67 G144D RP	GCCGACGCCGCAATCAAGCAGGATCCT
Lr67 G144K FP	GCCGACGCCGCACTTAAGCAGGATCCTGCCGATGATGA
Lr67 G144K RP	TCATCATCGGCAGGATCCTGCTTAAGTGCGGCGTCGGC
Lr67 G144S FP	CCGACGCCGCAACTAAGCAGGATCCTG
Lr67 G144S RP	CAGGATCCTGCTTAGTTGCGGCGTCGG
Lr67 G144W FP	CGACGCCGCACCAAAGCAGGATCCTGCCGATG
Lr67 G144W RP	CATCGGCAGGATCCTGCTTTGGTGCGGCGTCG
AtSTP13 G145R FP	CAGGCAGGATATTGCTTCGTTGTGGAGTTGGGTTC
AtSTP13 G145R RP	GAACCCAACTCCACAACGAAGCAATATCCTGCCTG
SbSTP13 G144R FP	GGATCCTGCTCCGCTGCGGCGTC
SbSTP13 G144R FP	GACGCCGCAGCGGAGCAGGATCC
GLUT1 G130S FP	GCCGCTTCATCATCAGTGTGTACTGCGGC
GLUT1 G130S RP	GCCGCAGTACACACTGATGATGAAGCGGC
Genotyping (KASP markers)	
HvSTP13^{G144} FP	GAAGGTCGGAGTCAACGGATTCATCGTCGGCAGGATCCTGCTCG
HvSTP13^{R144} FP	GAAGGTGACCAAGTTCATGCTCATCGTCGGCAGGATCCTGCTCC
HvSTP13 RP	CGGTGTCTTTTGCTTACTGG
Lr67sus FP	GAAGGTCGGAGTCAACGGATTTCATCATCGGCAGGATCCTGCTTG
Lr67res FP	GAAGGTGACCAAGTTCATGCTTCATCATCGGCAGGATCCTGCTTC
Lr67 RP	AACGTACGTAATCTTGCTTACTGA

Supplementary Table S2. Amplified and synthesised fragments and constructs used in this study. p426ADH1, pDR195/pDR196 constructs were used for yeast experiments, pGEMHE-DEST constructs were used for oocyte experiments.

**Fragment**	**Obtained by**	**Vector backbone**
Lr67sus	As described (p426ADH1; Moore et al., 2015), (Milne, Dibley et al., 2023)	p426ADH1, pDR196, pDR196T,
Lr67res	As described (p426ADH1; Moore et al., 2015), (Milne, Dibley et al., 2023)	p426ADH1, pDR196, pDR196T,
Lr67res C75Y	As described (Milne, Dibley et al., 2023)	pDR196
Lr67res E160K	As described (Milne, Dibley et al., 2023)	pDR196
Lr67res G208D	As described (Milne, Dibley et al., 2023)	pDR196
Lr67res G217D	As described (Milne, Dibley et al., 2023)	pDR196
Lr67sus G144A	Site-directed mutagenesis	pDR196
Lr67sus G144C	Site-directed mutagenesis	pDR196
Lr67sus G144D	Site-directed mutagenesis	pDR196
Lr67sus G144E	Synthesised and sub-cloned by Twist	pDR196
Lr67sus G144K	Site-directed mutagenesis	pDR196
Lr67sus G144S	Site-directed mutagenesis	pDR196
Lr67sus G144W	Site-directed mutagenesis	pDR196
FLAG-Lr67sus	Sub-cloned from p426ADH1 (Moore *et al.*, 2015)	pDR196
FLAG-Lr67res	Sub-cloned from p426ADH1 (Moore *et al.*, 2015)	pDR196
HvSTP13	As described (Milne *et al.*, 2019)	pDR196
HvSTP13 G144R	As described (Milne *et al.*, 2019)	pDR196
HvSTP13 G144R, V387L	As described (Milne *et al.*, 2019)	pDR196
AtSTP13	Synthesised by IDT	pDR196
AtSTP13 G144R	Site-directed mutagenesis	pDR196
SbSTP13	Synthesised by GeneArt	pDR196
SbSTP13 G144R	Site-directed mutagenesis	pDR196
XylE	Synthesised by IDT	pDR196
XylE G137R	Site-directed mutagenesis	pDR196
GLUT1	Synthesised by GeneArt	pDR195
GLUT1 G130R	Synthesised by GeneArt	pDR195
GLUT1 G130S	Site-directed mutagenesis	pDR195
TmClC-0	Synthesised by IDT	pDR196
AtHKT1	Synthesised by IDT	pDR196
Lr34sus	As described (Milne, Dibley et al., 2023)	pDR195
Lr34res	As described (Milne, Dibley et al., 2023)	pDR195
Lr67res genomic fragment	As described (Moore et al., 2015)	pVecNeo

HvSTP13^{G144R,V387L} genomic fragment	Synthesised by Epoch Biolabs	pWBVec8 binary vector

REFERENCES

Milne RJ, Dibley KE, Schnippenkoetter WH, Mascher M, Lui AC, Wang L, Lo C, Ashton AR, Ryan PR, Lagudah E. 2019. The wheat *Lr67* gene from the Sugar Transport Protein 13 family confers multipathogen resistance in barley. Plant Physiology 179, 1285-1297.

Moore JW, Herrera-Foessel S, Lan C, et al. 2015. A recently evolved hexose transporter variant confers resistance to multiple pathogens in wheat. Nature Genetics 47, 1494-1498.
